# Supplementary material for: Different historical generation intervals in human populations inferred from Neanderthal fragment lengths and mutation signatures
Source: Nat Commun. 2021 Sep 7;12:5317. doi: 10.1038/s41467-021-25524-4 (PMC8423828; doi:10.1038/s41467-021-25524-4)
Supplement: Supplementary file 1 — Supplementary Information [file 41467_2021_25524_MOESM1_ESM.pdf]

Different historical generation intervals in human populations inferred from Neanderthal fragment lengths and mutation signatures

Moisès Coll Macià[1*], Laurits Skov[2], Benjamin Marco Peter[2] and Mikkel Heide Schierup[1*]

1. Bioinformatics Research Centre, Aarhus University, Aarhus C, Denmark
2. Max Planck Institute for Evolutionary Anthropology, Leipzig, Germany

These authors contributed equally: Moisès Coll Macià and Laurits Skov

* Corresponding authors: moicoll@birc.au.dk, mheide@birc.au.dk

Supplementary Information

S1 - Identification of archaic fragments in non-African individuals extant populations and ancient samples ... 3

Ancient samples ... 4

Training the Hidden Markov model and decoding archaic fragments in each sample ... 4

Supplementary Figure 1 ... 6

Supplementary Table 1 ... 7

S2 - Archaic fragment length gradient around the world is consistent with using other quantifications and filters ... 8

Supplementary Figure 2 ... 10

S3 - Archaic fragment summary statistics per individual per region in extant populations and ancient samples ... 11

Supplementary Table 2 ... 11

Supplementary Table 3 ... 11

S4 - Population-specific recombination maps do not explain differences in archaic fragment length distributions ... 12

Supplementary Figure 3 ... 12

Supplementary Figure 4 ... 13

Supplementary Table 4 ... 14

S5 - Differences on archaic fragments between West Eurasia and East Asia regions are replicated in the population level comparison ... 15

Supplementary Figure 5 ... 17

Supplementary Figure 6 ... 18

Supplementary Table 5 ... 19

S6 - West Eurasia and East Asia fragment comparisons of archaic fragment genomic coverage ... 20

Supplementary Figure 7 22
Supplementary Figure 8 23
Supplementary Table 6 24
Supplementary Table 7 24
Supplementary Table 8 25
S7 - Simulations support a single Neanderthal pulse to the ancestors of East Asia and West Eurasia 26
Supplementary Figure 9 28
Supplementary Figure 10 29
Supplementary Figure 11 30
S8 - Derived alleles call outside regions with evidence of archaic introgression and acquired after the Out-of-Africa in SGDP samples 31
Supplementary Figure 12 34
Supplementary Table 9 35
Supplementary Table 10 37
S9 - Estimation of the different parental generation time in West Eurasia and East Asia 38
Supplementary Figure 13 40
S10 - Mutation spectrum correlation with mean parental age and potential bias due to difference in mean paternal and maternal age in de deCODE dataset 41
Supplementary Figure 14 42
Supplementary Figure 15 43
Supplementary Figure 16 44
Supplementary Table 11 46
S11 - Sex Specific mutational patterns 47
X-to-A ratio 47
C>G maternaly enriched regions 47
Y chromosome 48
Supplementary Figure 17 50
Supplementary Table 12 51
Supplementary Table 13 51
S12 - Source Data 52
References 54

S1 - Identification of archaic fragments in non-African individuals extant populations and ancient samples

We call archaic fragments in the samples of the Simons Genome Diversity Project (SGDP)[1] and ancient samples analysed in this study as described in Skov et al. 2020 and 2018[2,3] - a step by step tutorial is also available at https://github.com/LauritsSkov/Introgression-detection.

The method is described generally in the Methods section. In this section, we describe the specifics of the pipeline used in this study.

<u>Outgroup variants set, window mutation rate and callability and derived allele polarization for the SGDP dataset</u>

To generate the set of variants seen in the outgroup, we merged all variants from the following populations:

1. All Sub-Saharan Africans (populations: YRI, MSL, ESN) from the 1000 Genomes Project (1KGP)[4] and
2. All Sub-Saharan African populations from SGDP (this excludes Sharawi and Mozabite populations from the African supergroup) [1] except individuals from the Masai and Somali populations because they are reported to have some West Eurasian genetic component.

We determine the background mutation rate as the SNP density in the outgroup samples in windows of 100 kb.

To generate the callability regions, we merged the following files:

1. The 1KGP Callability file (hg19)

ftp://ftp.1000genomes.ebi.ac.uk/vol1/ftp/release/20130502/supporting/accessible_genome_masks/StrictMask/

2. Repeatmasker file (hg19)

ftp://hgdownload.cse.ucsc.edu/goldenpath/hg19/bigZips/chromFaMasked.tar.gz

To polarize alleles into ancestral and derived alleles we used the following file:

http://web.corral.tacc.utexas.edu/WGSAdownload/resources/human_ancestor_GRCh37_e71/

Ancient samples

We also call archaic fragments in 7 ancient samples (Supplementary Table 1) where the genome wide coverage is > 10X in order to genotype them reliably. In addition to the callability filter used above, we also require reads to map in regions where more than 50% of 31-kmers map uniquely - the track can be downloaded from:
https://bioinf.eva.mpg.de/map35/50/

For each sample, we mask the terminal 5 bases both in the 5' and 3' ends of the read to minimize ancient DNA damage. We also only consider reads without indels.
We call variants using samtools mpileup[5] (version 1.12), taking the frequency of known alleles from the 1KGP into account (see `--prior-freqs` command from http://samtools.github.io/bcftools/bcftools.html). We keep variants that have a quality score > 50.

For each sample, we count the number of derived alleles not found in the outgroup and calculate the transition/transversion ratio (Ts/Tv ratio, Supplementary Table 1). In contemporary humans this value is around 2. We note that despite our filtering, Anzick1 has a much higher Ts/Tv ratio and we therefore discard it for further analysis leaving us with 6 samples.

Training the Hidden Markov model and decoding archaic fragments in each sample

For each extant non-African individual from the SGDP and the ancient samples, we filtered out all sites where the derived variant is found in our outgroup population and sites that are not in our callable regions.

Then, for the extant individuals, we train the HMM and find the best fitting emission and transition values.

The enrichment of transitions observed in ancient individuals, especially Stuttgart, can lead to wrong parameter estimation when we train our HMM. To account for this, we fixed the HMM parameters for all samples with the following values:

```
states = ["Human", "Archaic"]
starting_probabilities = [0.98, 0.02]
transitions = [[0.9995,0.0005],[0.012,0.98]]
emissions = [0.040, 0.35]
```

The starting probabilities are fixed assuming a 2% Neanderthal sequence content in the samples. The transition and emission probabilities are obtained by training the hmm parameters using the 1KGP data as shown in https://github.com/LauritsSkov/Introgression-detection. We do not train the parameters with the ancient samples due to ancient DNA damage influencing these.

Finally, we identified tracks of archaic introgression in the whole genome of each individual. Archaic fragments for the individuals of the SGDP data set and ancient samples are provided in Data1_archaicfragments.txt. The archaic fragments in the ancient samples are visualized in Supplementary Figure 1.

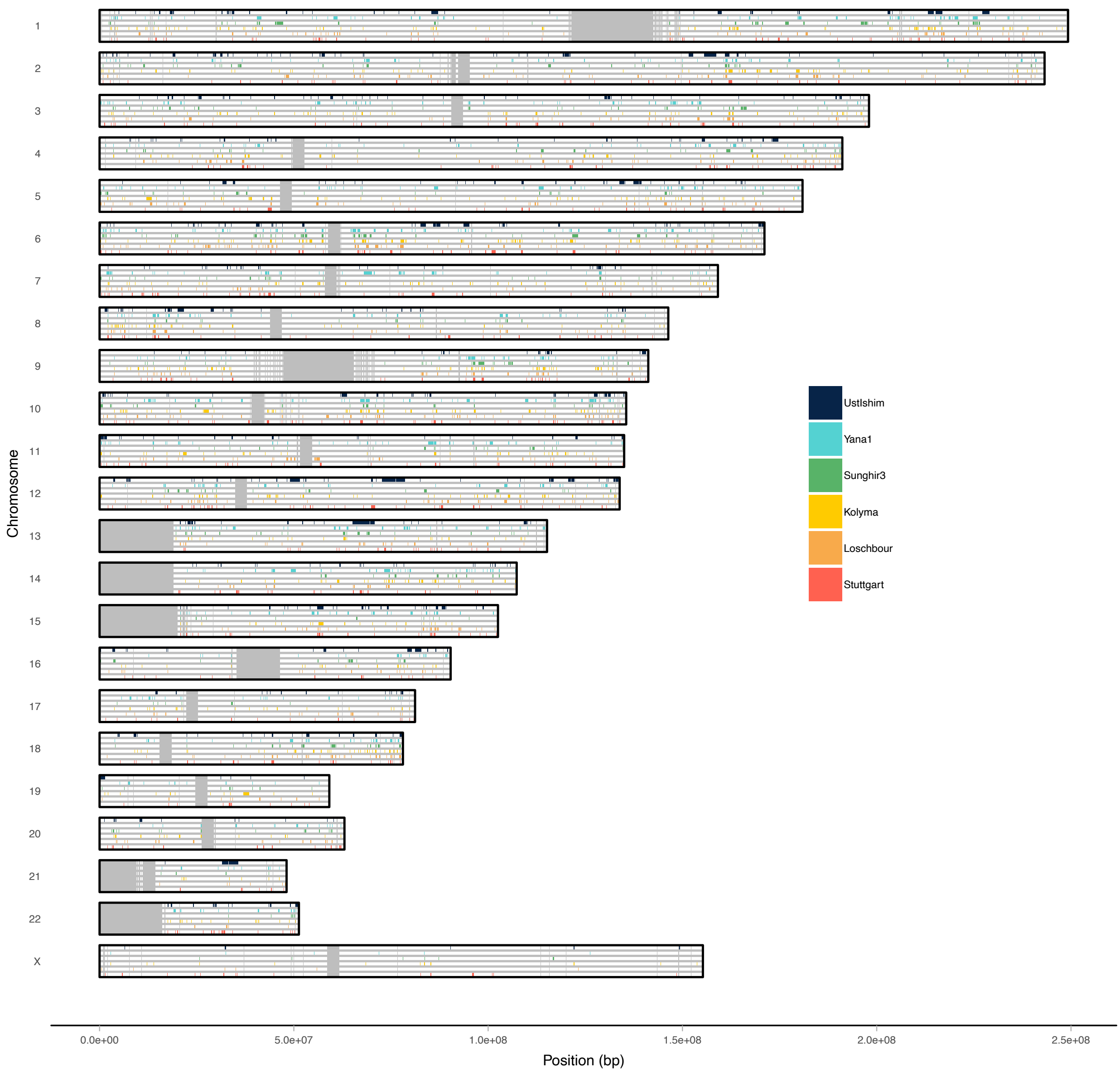

Supplementary Figure 1. Archaic fragments in Ust'-Ishim, Loschbour and Stuttgart ancient samples. Each horizontal rectangle represents a chromosome (hg19). In each chromosome, it is shown the archaic fragments found in Ust'-Ishim, Loschbour and Stuttgart ancient samples (colour coded). Wide grey bands on the chromosomes show the non-callable portions of the genome (hg19).

Sample	Reference	Age	Coverage	Called bp	Ts	Tv	Ts+Tv	Ts/Tv
Ust'-Ishim	Fu et al. 2014[6]	45,000	37.4	1,222,190,614	33,744	17,531	51,275	1.92
Yana1	Sikora et al. 2019[7]	39,000	26.3	1,114,080,903	38,540	18,447	56,987	2.09
Sunghir3	Sikora et al. 2017[8]	34,000	10.75	1,245,732,121	26,773	13,500	40,273	1.98
Anzick1	Rasmussen et al. 2014[9]	13,000	14.4	1,144,597,852	38,223	14,804	53,027	2.58
Kolyma	Sikora et al. 2019[7]	10,000	15.3	1,206,453,295	41,704	20,400	62,104	2.04
Loschbour	Lazaridis et al. 2014[10]	8,000	19.9	1,241,269,102	40,777	19,211	59,988	2.12
Stuttgart	Lazaridis et al. 2014[10]	7,000	18.1	1,243,802,585	41,796	19,244	61,040	2.17

Supplementary Table 1. Sequencing and quality statistics for the 7 human ancient samples. Anzick1 has been highlighted due to its high Ts/Tv ratio.

S2 - Archaic fragment length gradient around the world is consistent with using other quantifications and filters

We studied the robustness of the difference in mean archaic fragment length among the 5 geographical groups studied applying multiple filters.

1) Median instead of mean

The mean is very sensitive to outliers. In our case, very long archaic fragments, for example in East Asians, could increase the mean and thus show an unrealistic pattern among regions. To avoid that, we use median instead because it is more robust to outliers.

2) Vindija genome-like fragments

The method used in this study is able to find archaic fragments whose variation is not fully captured by the sequenced archaic individuals[2]. The difference in archaic fragment length can potentially be affected if there is a distinct archaic content among the extant populations studied here - for example, a greater and more recent Denisova component in Asia[11].

It is known that the majority of the archaic component in Eurasia and America is from a Neanderthal population closely related to the Vindija genome[12]. Thus, we restrict fragments used in this analysis to share more variation with the Vindija Neanderthal genome than the Altai Neanderthal genome or the Denisovan genome.

3) High confidence archaic fragments

The method used in this study, returns the archaic fragments found in a genome with an associated mean posterior probability. We restricted archaic fragments compared to be of a high confidence (mean posterior probability >= 0.9).

When we study the archaic fragment difference among individuals in Eurasia and America applying the three different types of filters explained above, we can see that the pattern observed using all fragments holds (Supplementary Figure 2). We conclude that the difference in archaic fragment length is genuine and not depending on the factors exposed above.

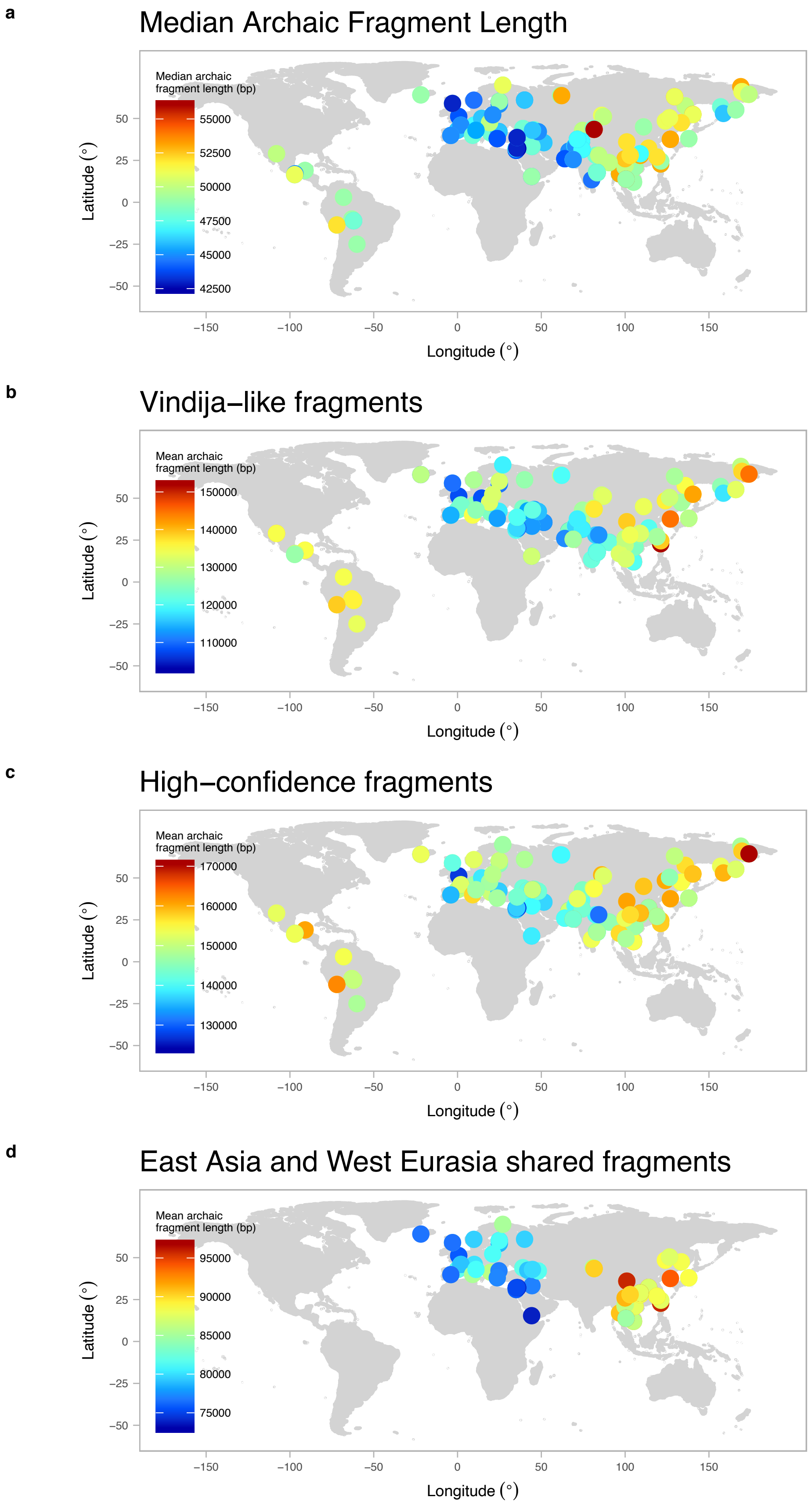
a
Median Archaic Fragment Length
Median archaic fragment length (bp)
55000
52500
50000
47500
45000
42500
Latitude (°)
50
25
0
−25
−50
−150
−100
−50
0
50
100
150
Longitude (°)
b
Vindija–like fragments
Mean archaic fragment length (bp)
150000
140000
130000
120000
110000
Latitude (°)
Longitude (°)
c
High–confidence fragments
Mean archaic fragment length (bp)
170000
160000
150000
140000
130000
Latitude (°)
Longitude (°)
d
East Asia and West Eurasia shared fragments
Mean archaic fragment length (bp)
95000
90000
85000
80000
75000
Latitude (°)
Longitude (°)

Supplementary Figure 2. Archaic fragment length distribution around the world with specific filters. World map (Methods) showing as circles the samples from SGDP used in this study coloured according to the mean or median average archaic fragment length applying filters to the data. **a)** Median archaic fragment length is plotted instead of the mean. **b)** Only fragments with more SNPs shared with the Vindjia genome than the Denisova or the Altai genomes are used. **c)** Only high confidence archaic fragments (posterior probability >= 90%) are used. **d)** Only shared individual fragments (Supplementary Figure 7, S6) between East Asians and West Eurasians.

S3 - Archaic fragment summary statistics per individual per region in extant populations and ancient samples

Region	Number of samples	Number archaic fragments		Archaic seq (bp)		Mean archaic fragment length (bp)	
		mean	SE	mean	SE	mean	SE
West Eurasia	71	980.97	6.94	72,129,573.79	745,671.83	73,449.23	373.07
South Asia	39	1,123.00	11.25	84,566,166.34	1,130,742.30	75,221.84	411.09
America	20	1,078.84	10.22	86,324,786.21	806,781.45	80,058.47	563.05
Central Asia Siberia	27	1,133.26	8.83	92,428,433.59	939,993.02	81,543.88	459.91
East Asia	45	1,161.59	6.70	95,548,011.13	708,871.76	82,259.38	401.92

Supplementary Table 2. Archaic fragment summary statistics per individual per region. Summary statistics of the fragments found among the individuals of the 5 main regions. For each statistic, the mean and its SE (Methods) are provided.

Ancient samples	Number archaic fragments	Archaic seq (bp)	Archaic fragment length (bp)	
			mean	SE
Ust'-Ishim	646	121,078,000	187,390.83	12,363.51
Yana1	847	115,006,000	135,785.00	6,229.82
Sunghir3	593	67,174,000	113,280.88	5,082.35
Kolyma	948	87,830,000	92,673.62	3,982.94
Loschbour	802	76,115,000	94,918.94	3,675.41
Stuttgart	755	65,449,000	86,681.36	3,812.45

Supplementary Table 3. Archaic fragment summary statistics per ancient sample. Summary statistics of the fragments found in the three ancient samples. For the archaic fragment length, the mean and its SE (Methods) are provided.

S4 - Population-specific recombination maps do not explain differences in archaic fragment length distributions

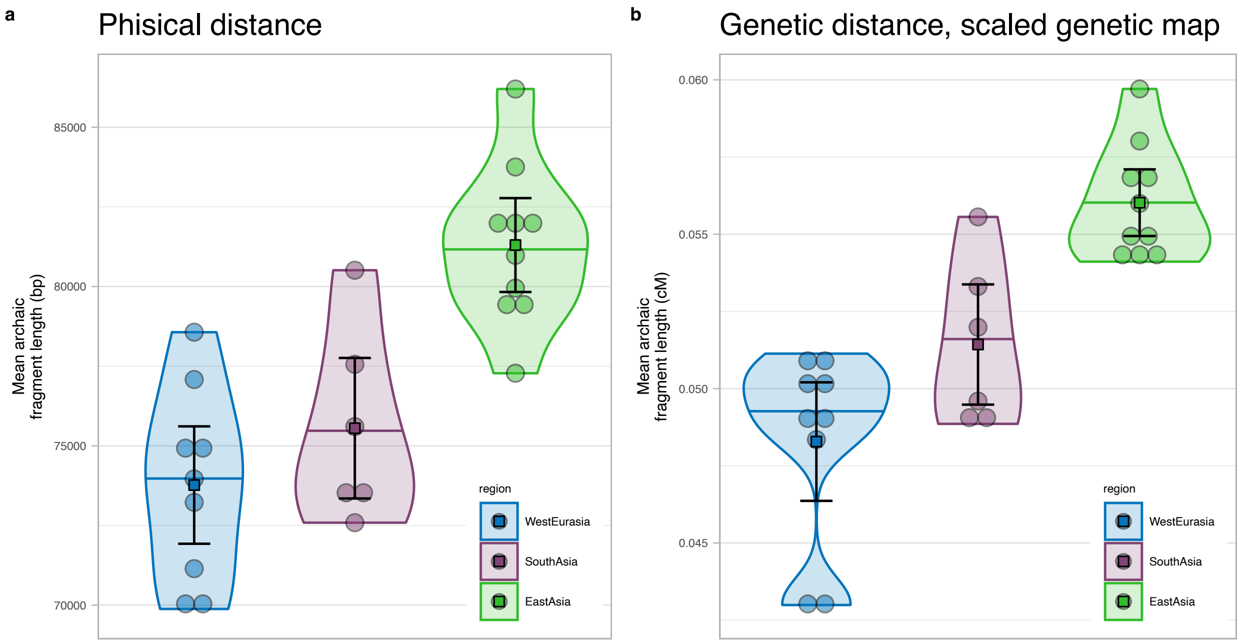

Supplementary Figure 3. **Mean archaic fragment length distributions in physical and genetic distances**. Distributions per region (colour coded) are shown violin plots. Individual values are shown as dots. The median is shown as a horizontal line in each violin plot. The mean and its 95%CI of each distribution are shown as a coloured square with their corresponding error bars. **a)** Physical distance (bp). **b)** Genetic distance (cM) corrected by shortest chromosome length. The sample sizes of individuals for each region for which summary statistics are derived from are indicated in Supplementary Table 4.

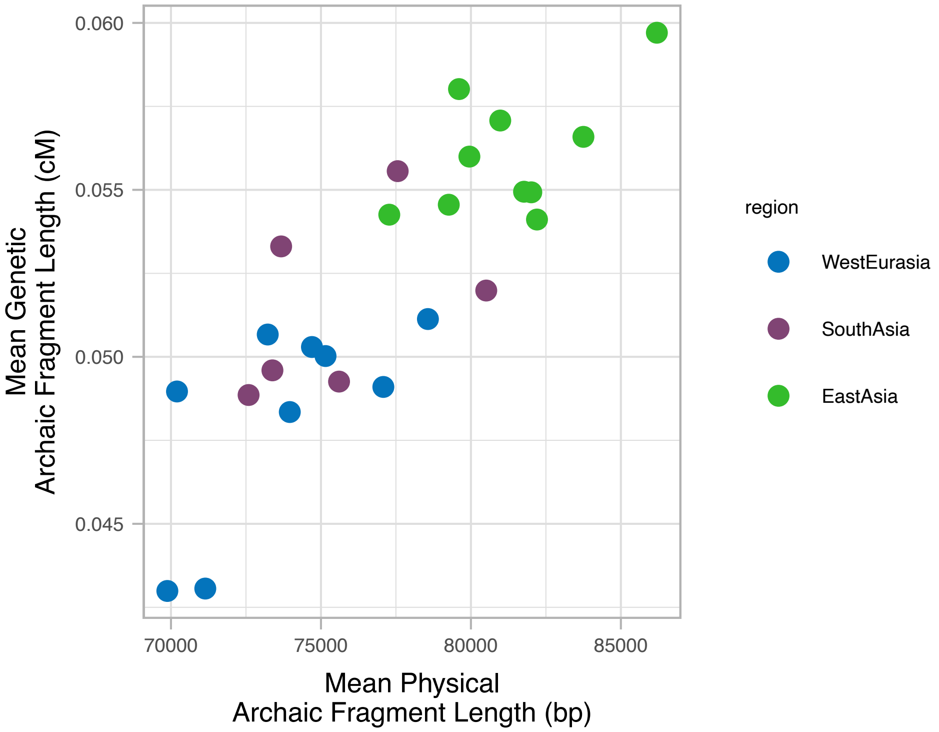

Supplementary Figure 4. **Mean archaic fragment length per individual correlations between physical and genetic distances**. Individual values are shown as dots, coloured depending on the region they belong to.

Region in SGDP	N samples in SGDP	SGDP population	1KGP population
East Asia	3	Dai	CDX
	2	Han	CHB
	3	Japanese	JPT
	2	Kinh	KHV
West Eurasia	3	Finnish	FIN
	2	English	GBR
	2	Spanish	IBS
	2	Tuscan	TSI
South Asia	2	Bengali	BEB
	4	Punjabi	PJL

Supplementary Table 4. Non-African population correspondence between SGDP data set and 1KGP data set.

S5 - Differences on archaic fragments between West Eurasia and East Asia regions are replicated in the population level comparison

In our analysis, we divide the non-African individuals of the SGDP data into 5 main regions to compare them in terms of archaic fragment number, length and archaic sequence. In this section, we use complementary data to investigate whether similar differences are found if we use larger samples from more homogeneous populations to test for differences in archaic fragment statistics between West Eurasia and East Asia. This both serves as confirmation of the original observations in an independent data set with different variant calling and a test of whether the pooling of individuals in SGDP into regions causes biases due to the individuals having different ancestry and perhaps different archaic fragment lengths.

We call archaic fragments in individuals from 2 populations of each region which are also represented in the Human Genome Diversity Project (HGDP)[13] panel (see how on *Archaic fragments call in HGDP populations* below). We chose this data set since the sample size per population is greater than in SGDP (Supplementary Table 5). We used four populations that satisfy the following criteria:

1. Populations with the greatest and smallest mean archaic fragment length in the SGDP data from the West Eurasia and East Asia regions respectively that are represented in HGDP (Supplementary Figure 5). These are Sardinians (mean archaic fragment length = 79,355 bp) and Lahu (mean archaic fragment length = 78,330 bp).
2. For both regions, we selected the population of SGDP with the greatest sample sizes in HGDP. These are Palestinians and Han Chinese.

The fragments of the individuals selected can be found in Data3_HGDParchaicfragments.txt.

The variance in West Eurasia and East Asia regions in the SGDP is similar to the four populations from the HGDP in all of the 3 statistics evaluated (Supplementary Table 5, Supplementary Figure 6). This indicates that the regional variance observed in the SGDP data is likely to stem primarily from intra-population variance, rather than inter-population variance.

We then compare, for each SGDP region, if the two representative populations from the HGDP data set have distinctive distributions of archaic fragment length (Supplementary Figure 6). While Lahu and Han people have similar distributions (P value = 0.71, permutation test, Methods), Sardinians have longer fragments than Palestinians (P value < 1e-5, permutation

test, Methods). This reflects the fact that West Eurasia is a heterogeneous group which incorporates populations with different histories[10,14,15] compared to East Asians, which seems to gather more homogeneous groups.

Finally, Sardinians and Lahu people - as representatives of West Eurasia and East Asia regions -, have also different fragment sizes (Supplementary Figure 6, P value = 2e-5, permutation test, Methods). This result shows that the difference observed between West Eurasia and East Asia is replicated in an independent data set with more homogeneous populations.

Archaic fragments call in HGDP populations

To call archaic fragments in the HGDP data we follow the methodology described in the Methods section and in S1. However, since the HGDP data is mapped to the GRCh38 reference genome, we modify certain steps to create the source files.

First, we use the following individuals to generate our outgroup:

1. All Sub-Saharan Africans (populations: YRI, MSL, ESN) from the new high-coverage 1KGP in GRCh38 coordinates [16] and
2. All Sub-Saharan African individuals from HGDP with less than 0.1% admixture signals from other continental populations (for individuals with substantial admixture inferred (>0.1%), this was majoritarily European). Admixture estimates were kindly provided by the corresponding authors of [13].

We determine the background mutation rate as the SNP density in the HGDP outgroup samples in windows of 100 kb.

The callability regions were extracted from the accessibility mask file included in the HGDP data set:

ftp://ngs.sanger.ac.uk/production/hgdp/hgdp_wgs.20190516/
production/hgdp/hgdp_wgs.20190516/accessibility-mask/hgdp_wgs.20190516.mask.bed

To polarize alleles into ancestral and derived alleles we used the field “AA_ensembl” in the HGDP VCFs, which corresponds to the Ensembl’s homo_sapiens_ancestor_GRCh38_e86 files.

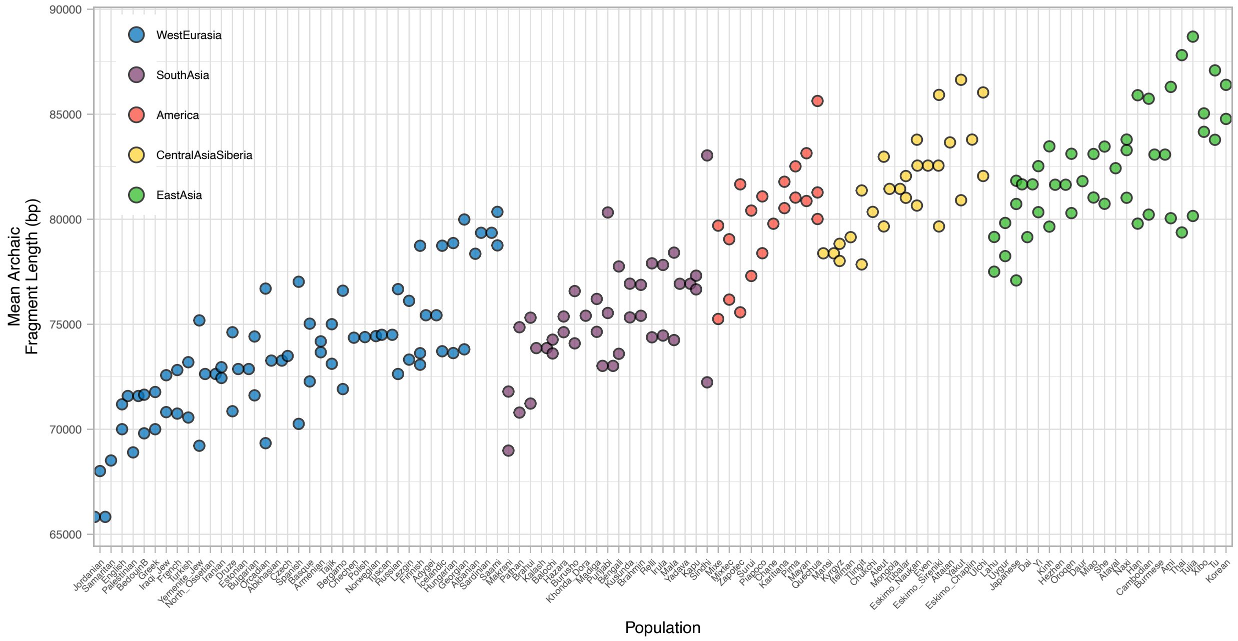

Supplementary Figure 5. Mean archaic fragment length per population. Individual values are shown as dots for each population of the five regions in SGDP data. Populations on the x-axis are sorted per region and ascending population average among all its individuals.

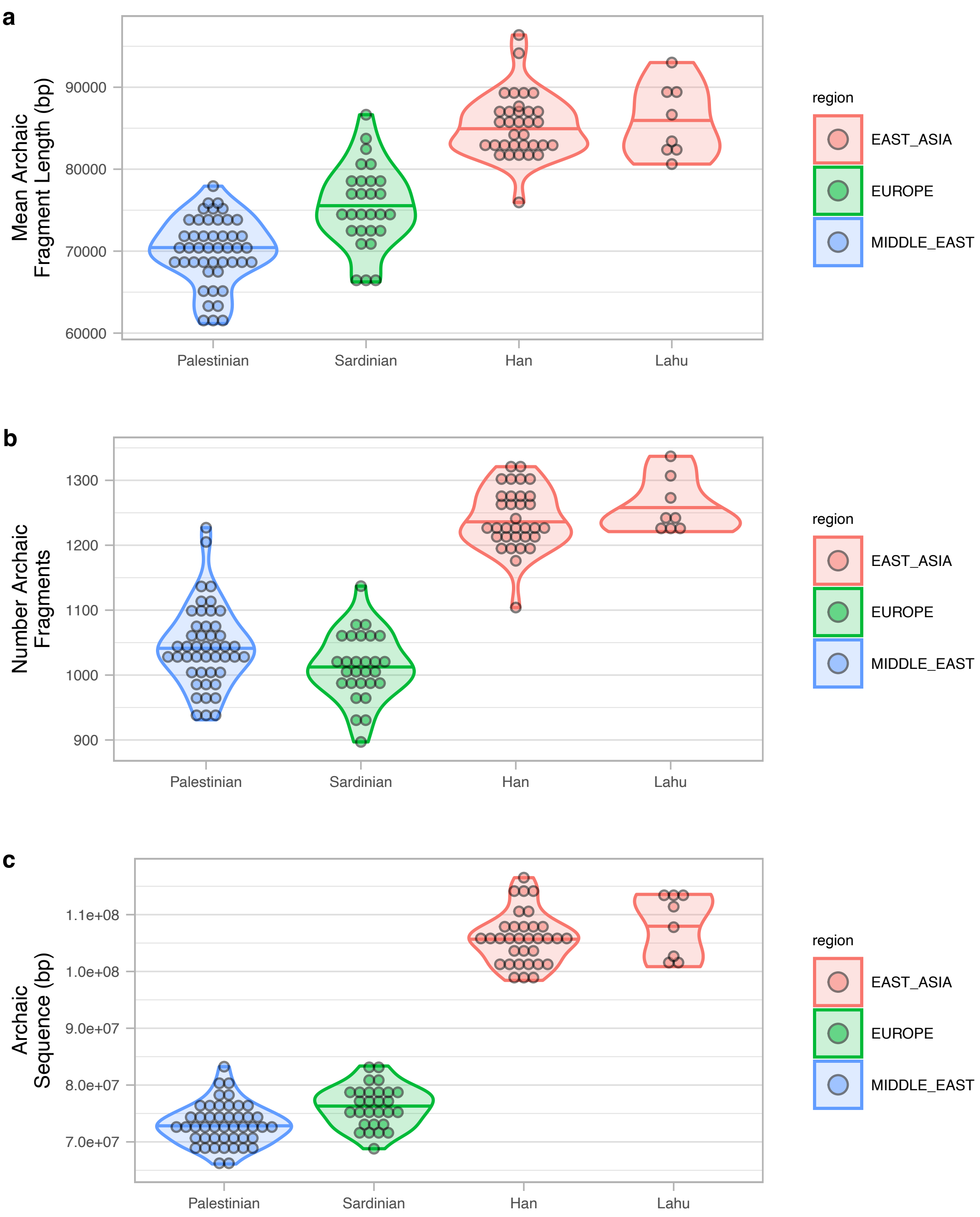

Supplementary Figure 6. **Archaic fragment statistics distributions for four populations of the HGDP data set representing West Eurasia and East Asia SGDP regions**. Distributions of different archaic fragment statistics per population (colour coded by HGDP region annotation) are shown as violin plots. Individual values are shown as dots. The median is shown as a horizontal line in each violin plot. Palestinians and Sardinians are representatives of the West Eurasia group and Han and Lahu of the East Asian group. **a)** Mean archaic fragment length distributions in base pairs (bp). **b)** Number of archaic fragments distribution. **c)** Archaic sequence in base pairs (bp).

Region / Population	Number of samples	Number archaic fragments		Archaic seq (bp)		Mean archaic fragment length (bp)	
		mean	SD	mean	SD	mean	SD
**SGDP**							
West Eurasia	71	980.93	58.97	72,133,394.37	6,332,052.22	73,450.20	3,174.57
East Asia	45	1,161.58	45.38	95,549,133.33	4,820,291.63	82,259.24	2,723.39
**HGDP**							
Palestina	46	1,044.15	62.61	73,011,543.48	3,644,923.32	70,076.65	4,032.19
Sardinia	28	1,013.18	51.66	76,288,571.43	3,624,998.66	75,467.91	4,985.07
Han	33	1,241.12	47.32	105,849,787.88	4,611,437.59	85,354.28	3,904.24
Lahu	8	1,259.38	42.70	108,136,625.00	5,480,052.06	85,907.76	4,395.20

Supplementary Table 5. Distribution archaic fragment summary statistics per SGDP regions and HGDP populations. Distribution summary statistics of the fragments found West Eurasia and East Asia regions of SGDP data and in four populations from the HGDP data.

S6 - West Eurasia and East Asia fragment comparisons of archaic fragment genomic coverage

The collapsed East Asian archaic sequence (916,369,000 bp) is 1,06 times larger than the collapsed West Eurasian archaic sequence (866,945,000 bp) and more than half of the sequence is shared between the two (485,255,000 bp, Supplementary Table 8). We partially attribute this difference to the fact that East Asians have a higher Denisova component than West Eurasians[11]. To study that we repeated the analysis above filtering archaic fragments in each individual (before collapsing) depending on which of the three archaic genomes (Vindija Neanderthal genome[12], Altai Neanderthal genome[17], Denisova genome[18]) share the most variants to (below), following the methods in Skov et al. 2020[3]. Some fragments do not share variants with any of the 3 sequenced archaic genomes, and thus we classify them as unknown. There are also instances in which an archaic fragment does not share more SNPs with one of the archaic genomes but multiple, so we can't classify the affinity of the fragments; these fragments are called ambiguous fragments.

1) Denisova fragments

We only include archaic fragments which share more variants to Denisova genome than any of the two Neanderthal genomes.

2) nonDenisova fragments

In this analysis we exclude fragments used above from all the fragments. Thus, we include Vindija-like, Altai-like, ambiguous and unknown.

3) Neanderthal fragments

We only include archaic fragments that share more variants with either the Altai Neanderthal or the Vindija Neanderthal genomes than the Denisova genome. Neanderthal ambiguous fragments, fragments that share the same number of SNPs with Vindija or Altai but this number is higher than what is shared with the Denisova, are also included.

All results for the different filters are shown in Supplementary Table 8. The Denisova content is 3 times greater in East Asia than in West Eurasia (Denisova fragments filter). When this unequal component is removed (non-Denisova fragments filter), we can see that the collapsed archaic sequence is very similar between the two regions.

The analysis was repeated with fragments that share more variation with Neanderthal than with Denisova (Neanderthal fragments). In this case, we observe a 1.07 fold higher Neanderthal content in the East Asian group. We attribute this to the fact that since West Eurasia archaic fragments tend to be shorter, they do not contain enough SNPs to classify them to the category that they belong to. Thus, they are going to be more often classified as unknown compared to fragments in East Asia. Furthermore, the ^{2} method has higher false negative rate with short fragments, which will artificially decrease the total number of fragments in that region.

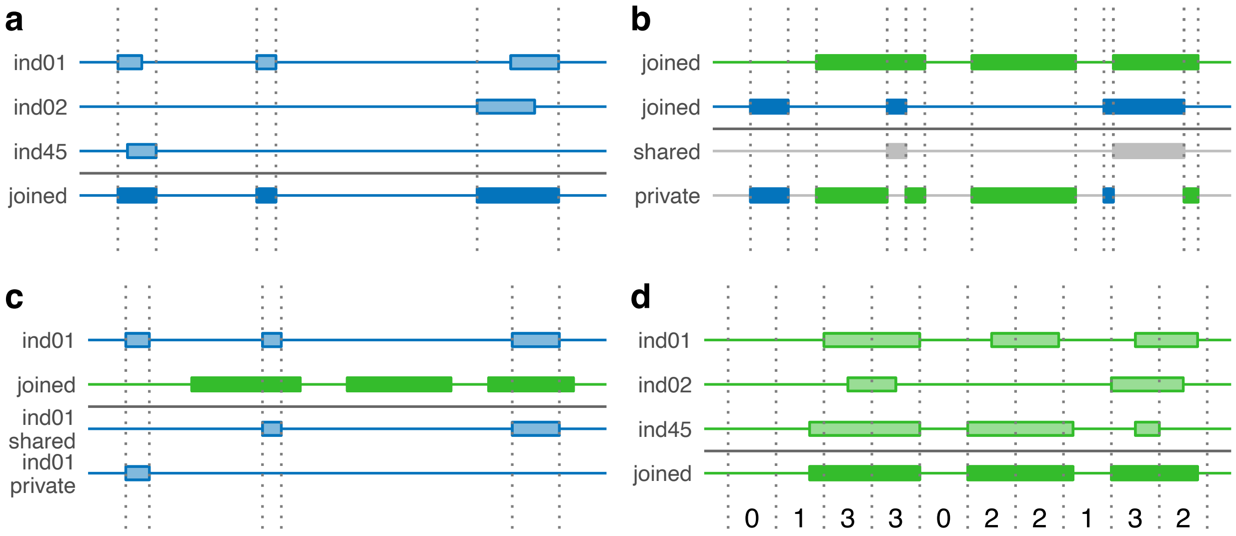

Supplementary Figure 7. West Eurasia and East Asia fragment comparison methods. Diagram showing the different methods to compare archaic fragments between West Eurasians and East Asians (Methods). Each horizontal line represents a genome. Wide bands on each genome represent archaic sequences. East Asia is represented in green colours and West Eurasia in blue. Grey colours are used when sequences are shared by both. Plain colours denote joined sequences and transparent colours show individual sequences. Vertical dashed lines are mainly used to point to genomic windows of interest. **a)** Joined region fragments. **b)** Shared and private joined region sequence. **c)** Shared and private individual fragments. **d)** Archaic frequency in 10 kb windows represented as the vertical grey lines intervals (note that in the main text, 1 kb windows are used instead).

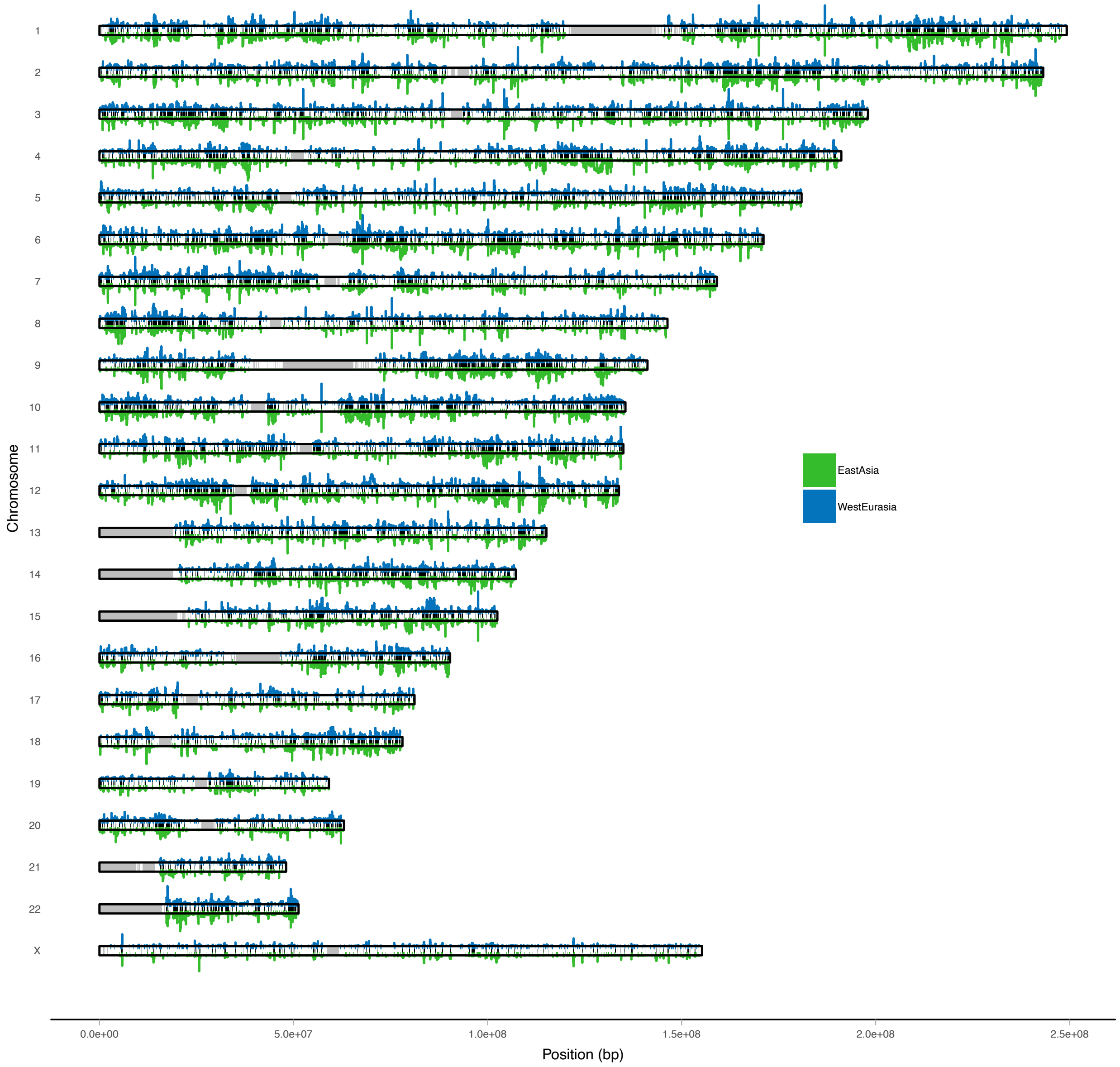

Supplementary Figure 8. The archaic landscape across the West Eurasian and East Asian genomes. Each horizontal rectangle represents a chromosome (hg19). In each chromosome, it is shown the joined region fragments for West Eurasia (blue upper bands) and East Asia (green lower bands). The shared joined region fragments are shown as black bands in the middle of each chromosome. For each region, the number of individuals that have an archaic fragment in a particular 1kb window are represented as lines (maximum number of individuals is 45 for each region). Grey bands on the chromosomes show the non-callable portions of the genome (hg19).

Region	Number of samples	Type	Archaic Sequence (kb)
West Eurasia	45	Shared	485,255 (55,97%)
		Private	381,690 (44,03%)
		All	866,945 (100%)
East Asia	45	Shared	485,255 (52,95%)
		Private	431,114 (47,05%)
		All	916,369 (100%)

Supplementary Table 6. Summary table of shared, private and total joined archaic sequence of West Eurasia and East Asia regions. Percent in respect of the total are shown in parenthesis.

Region	Number of samples	Type	Number archaic fragments		Archaic seq (bp)		Archaic fragment length (bp)	
			mean	SE	mean	SE	mean	SE
West Eurasia	45	Shared	756.20	9.44	59,878,285.67	956,290.19	79,061.50	479.81
		Private	221.56	2.86	11,476,262.63	241,069.15	51,736.34	738.84
		All	977.74	9.77	71,360,737.66	992,105.26	72,878.36	445.45
East Asia	45	Shared	913.80	5.09	81,726,409.34	586,299.59	89,450.85	477.09
		Private	247.79	3.76	13,828,084.06	250,216.66	55,783.23	572.33
		All	1,161.57	6.67	95,549,865.39	710,273.36	82,256.35	401.75

Supplementary Table 7. Summary statistics of the shared, private and total individual archaic fragments of West Eurasians and East Asians. For each statistic, the mean and its SE (Methods) are provided.

	Joined East Asia archaic sequence (kb)	Joined West Eurasia archaic sequence (kp)	Fold diff	Shared joined archaic sequence (kb)	East Asia shared (%)	West Eurasia shared (%)
All fragments	916,369	866,945	1.06	485,255	52.95	55.97
Denisova fragments	107,695	36,850	2.92	16,004	14.86	43.43
nonDenisova fragments	853,065	850,028	1.003	460,490	53.98	54.17
Neanderthal fragments	646,710	604,518	1.07	309,043	47.79	51.12

Supplementary Table 8. Joined archaic sequence in East Asia and West Eurasia and comparative statistics for different subsamples of archaic fragments (S6).

S7 - Simulations support a single Neanderthal pulse to the ancestors of East Asia and West Eurasia

In this study, we observe that East Asia individuals have longer and more archaic fragments compared to the rest of the world. This could be compatible with East Asians receiving archaic fragments from a much more recent Neanderthal admixture private to East Asia[19–21]. However, when East Asia and West Eurasia regions are compared by joining their fragments in each group, they seem to have similar amounts of total and shared archaic sequence.

In order to quantify the expected differences between populations in a scenario with a single Neanderthal pulse to the common ancestors of East Asians and West Eurasians (One Pulse) and another one with an additional and private pulse to East Asians (Two Pulses) (Supplementary Figure 9), we simulated whole genomes of both using `msprime`[22] as explained in the Methods section.

The private Neanderthal gene flow into the EA group in the second scenario is set to be around the time Neanderthals became extinct (38,000 ya)[23] in order to maximize the mean fragment length difference between EA and WE. Furthermore, the admixture proportion is ¼ of the first gene flow (0.5 % vs 2 %) to approximately match the difference in archaic content observed between West Eurasians and East Asians in the main text.

Supplementary Figure 10 shows the distributions of the mean archaic fragment length, number of fragments and total length per WE and EA and the comparison of their average values. While the Two Pulses scenario creates a noticeable increase in EA in terms of number of archaic fragments (~15%) and total archaic sequence (~20%), the increase in the mean archaic fragment length is much smaller (~2.5%) than observed in the analyses of SGDP genomes.

When we join fragments of both groups, similar to what we do for the SGDP data (Methods) and described in the Methods section, we observe similar abundance of shared archaic sequences for both groups in the One Pulse scenario (Supplementary Figure 11). However, in the Two Pulses scenario, there is a large decrease in the shared archaic sequence for the EA group that received the second gene flow.

Overall, we observe that the Two Pulses scenario, compared to the null model, provides EA group with an excess of private sequences (~58%, 8 points more than WE) and slightly increases the mean fragment length by ~2.5% compared to WE group. In contrast, the SGDP

samples from West Eurasia and East Asia show greater differences in terms of fragment length (~12%) and similar levels of total and shared sequence (53% and 56% respectively) as groups. Thus, we conclude that a second pulse scenario is not compatible with our observations.

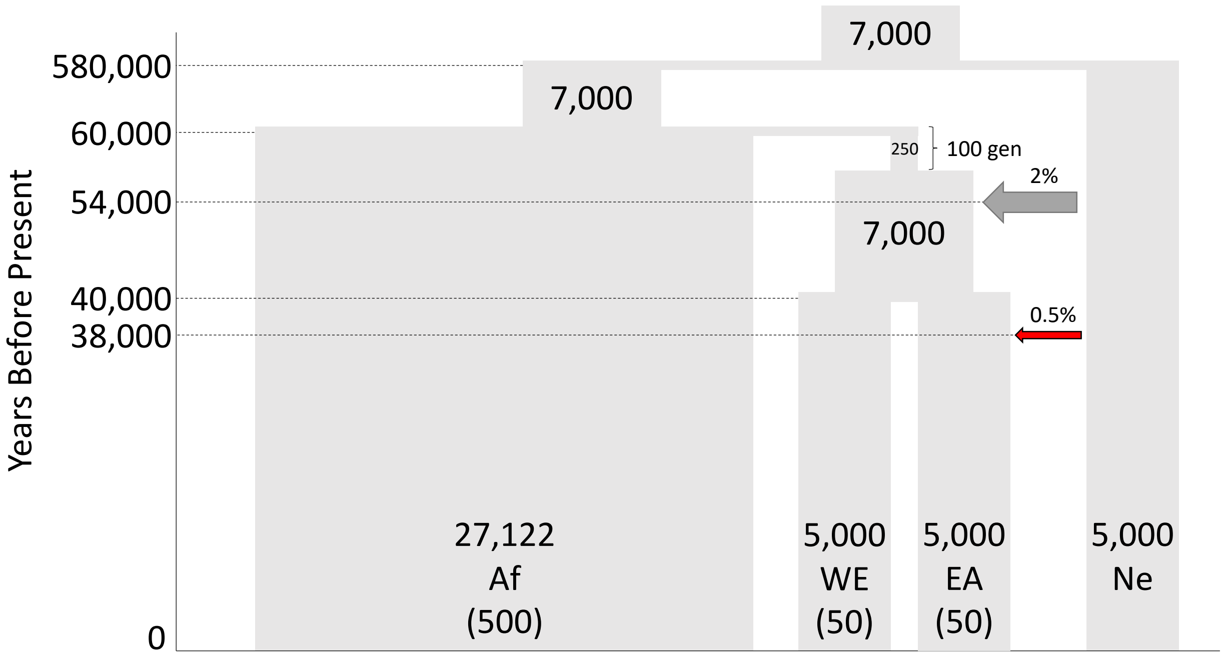

Supplementary Figure 9. One Pulse and Two Pulses demographic scenarios. Graphical representation of the demographic models simulated: One Pulse and Two Pulses models. Tree-like structures represent the phylogenetic relationships between the different groups (Af : Africa, WE : West Eurasia, EA : East Asia, Ne : Neanderthal). The width of each branch is scaled by the effective population size (also denoted in numbers). The timing of the events are shown on the y-axis in years besides the bottleneck of the WE and EA ancestral population which has a duration of 100 generations. The number of sampled diploid individuals for Af, WE and EA are shown in parenthesis. The difference between the two scenarios is the presence of the private Ne admixture to EA 38 ky (red arrow) in the Two Pulses model.

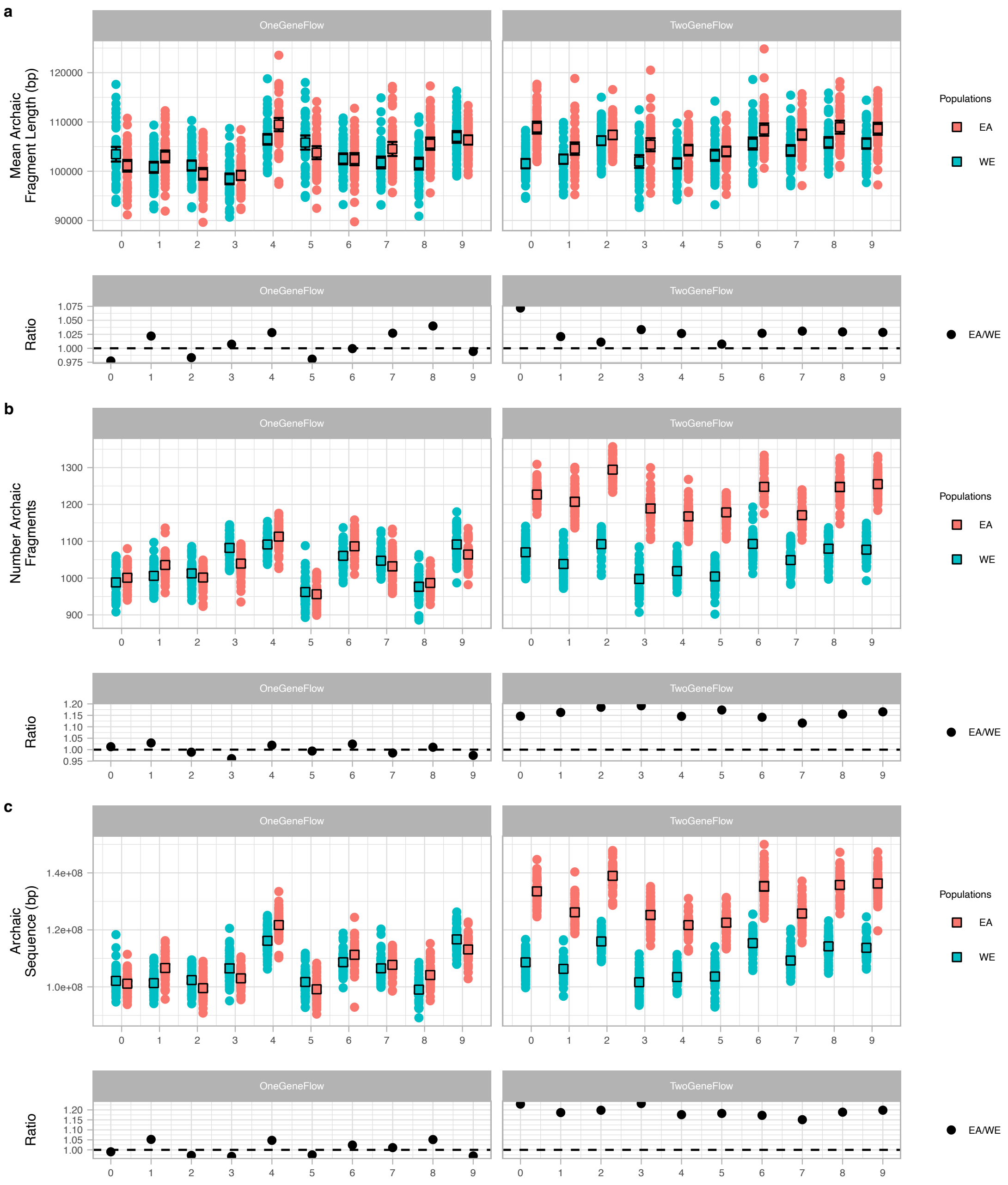

Supplementary Figure 10. Archaic fragment statistics distributions for the One Pulse and Two Pulses simulated scenarios. Archaic fragment length statistic distributions (first subpanel rows) for both groups (color coded) in the 10 replicates (x-axis) of each simulated scenario (subpanel columns). Values for each individual are shown as dots. Mean values for each distribution are shown as squares with their associated 95% CI as whiskers (computed as mean±(1.96*se); se = sd/sqrt(n)). Comparing the means of each distribution replicate, the ratio between EA and WE is shown (second subpanel rows) as black dots. **a)** Mean Archaic Fragment Length (bp) **b)** Number of archaic fragments **c)** Archaic sequence (bp).

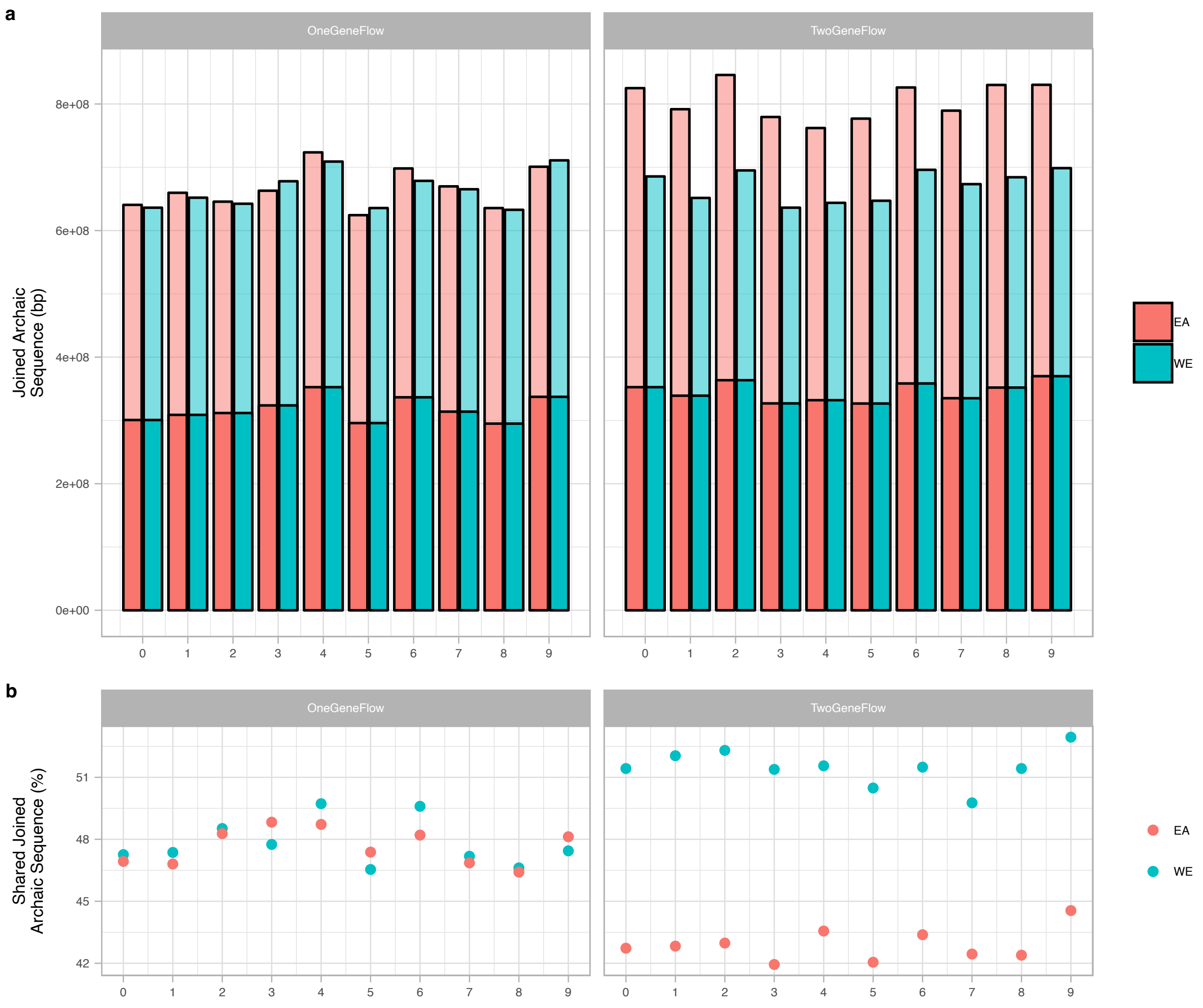

Supplementary Figure 11. Joined archaic fragments comparisons between WE and EA for the One Pulse and Two Pulses simulated scenarios. a) Joined archaic fragment length for each group (color coded) as bar plots in the 10 replicates (x-axis) of each simulated scenario (subpanels). Each bar is divided into shared (plain colour) and private sequence (transparent colour). **b)** For each replicate in **a)**, the percentage of shared sequence between the EA and WE are shown as points per population (colour coded).

S8 - Derived alleles call outside regions with evidence of archaic introgression and acquired after the Out-of-Africa in SGDP samples

We retrieved the genotypes of all polymorphic loci for each individual in the 5 main regions and African samples as explained in the Methods section. We masked repetitive regions and regions of the genome in which there is some evidence of archaic introgression in the following way:

1) Neandertal introgressed regions

Neanderthals had a different mutation profile than modern humans[3]. Thus, differences in Neanderthal content per individual could influence those analyses that explore the mutation spectrum differences among populations. Also, by removing these regions, we will base the mutation analysis on regions of the genome that we haven't explored in the archaic fragment length part of the study. Thus, the tests are going to be independent of each other.

To do that, we disregarded any polymorphism localized in a region with evidence of archaic introgression in any of the individuals analyzed in this study (Methods, S1). For that, we joined all archaic fragments called in any individual included in this study using this command:

```
bedtools merge -i ind1.bed ind2.bed … indN.bed > joined.bed
```

where `N` denotes the total number of individuals.

In total, the joined archaic region adds up to 1,632,776,000 bp.

2) Repeats

We also excluded repetitive regions in which sequencing errors are expected to be more prevalent. For that, we downloaded the human reference genome by using the following command:

```
for chr in `seq 1 22` X Y;
do
rsync -avzP
rsync://hgdownload.cse.ucsc.edu/goldenPath/hg19/chromosomes/chr${chr}.fa.gz .;
done
```

from which we created a bed file with the coordinates of the repeats from RepeatMasker and Tandem Repeats Finder (represented in the reference genomes fastas as lowercase letters in the fasta file).

These regions add up to 1,431,504,380 bp in total.

The intersection between the repetitive regions and the archaic regions correspond to 806,042,777 bp, which corresponds to 56.31% of the total repetitive regions sequence and 49.37% of the archaic sequence. Together, these regions add up to 2,258,237,603 bp. If we consider only the callable fraction - instead of the total genomic length of 3,036,303,846 bp - of the human genome (2,835,673,565 bp), 577,435,962 bp remain after masking by archaic and repetitive regions (20.36%).

Other filters on the SNP level were imposed for each polymorphism:

1) The SNP must be biallelic
2) The contiguous 5' and 3' base pairs of the focal SNP (context) must be called in the human reference genome (hg19)
3) 20% of the individuals must have the genotype for that SNP to be called
4) The chimpanzee reference genome in human coordinates must have the homologous base pair called for that position
5) No Sub-Saharan African (which excludes S_Mozabite-1, S_Mozabite-2, S_Saharawi-1 and S_Saharawi-2 samples from the African supergroup) samples can have the derived allele

The latter filter ensures that the polymorphisms investigated most probably arose after the Out-of-Africa expansion. S_Masai-1, S_Masai-2 and S_Somali-1 samples are not included in the Sub-Saharan African group because they are reported to have some West Eurasian genetic component in Mallick et al. 2016[1], which would affect our results. If African genomes with West Eurasian components are included in the African set, then, by the 5) filter, we are more likely to remove derived alleles private to West Eurasia than other regions.

Homozygous locus for the derived allele count as 2 mutations and heterozygous sites count as 1 for a given individual. The distribution of derived allele accumulation per region is shown in Figure 3 and the mean derived allele accumulation counts per region are provided in Supplementary Table 9.

Finally, we classified loci in different mutation types depending on the derived allele nucleotide, the ancestral allele nucleotide and their 5' and 3' nucleotide context. For example, as shown by the diagram below, a derived allele T that had an ancestral allele C with the

context G and A (5' and 3' respectively) would be denoted as GCA>T. Because we do not make distinction of the strand in which the mutation occurred, we collapsed strand-symmetric mutations. This is the same as saying that GCA>T is equivalent to TGC>A. This way, we end up with 96 mutation types.

```
    T
    *
5'-GCA-3'
3'-CGT-5'
    *
    A
```

Data2_mutationspectrum.txt provides the resulting counts of each individual for each mutation type in each chromosome.

The mutation types investigated in this study are 9:

- 6 mutation types in which only the ancestral and derived allele nucleotides were taken into account and C and T were used as ancestral (T>A, T>C, T>G, C>A, C>T, C>G)
- C>T mutations were further divided into 3 mutation types:
    - CpG>TpG mutations which are shown to evolve in a more clock-like manner[24].
    - TCC>TTC mutations which are in excess in Europeans compared to other human populations[25,26].
    - C>T' mutations which contain the rest of C>T mutations not included in the previous 2 types.

The distribution of derived allele accumulation per region is shown in Supplementary Figure 12 and the mean derived allele accumulation counts per region are provided in Supplementary Table 10.

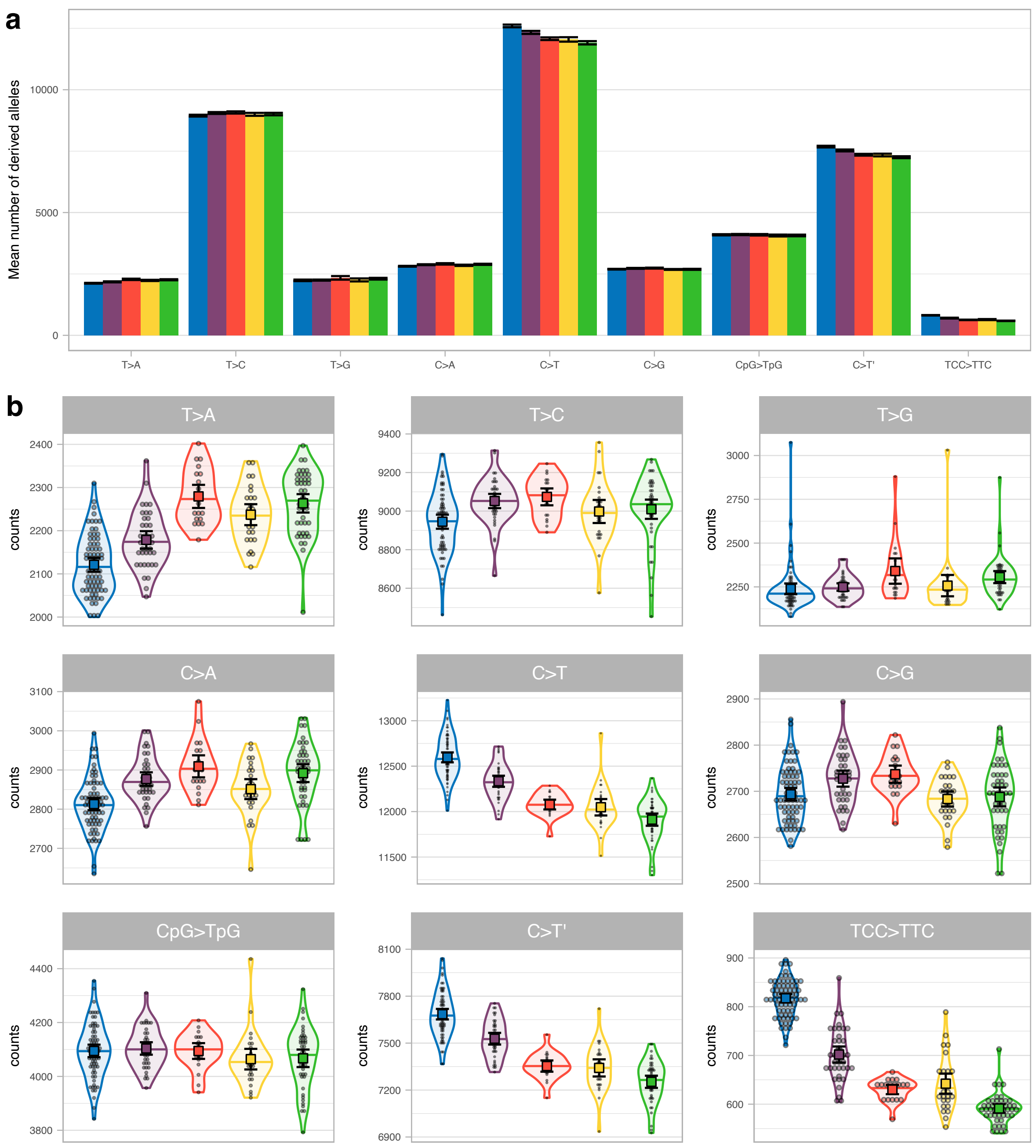

Supplementary Figure 12. Mean derived allele accumulation of the 9-mutation types per region. a) The mean number of derived alleles of each mutation type accumulated among individuals of the 5 regions (colour coded). The bar plot emphasises a comparison among mutation types. The 95%CI of each mean is shown as error bars. **b)** The same information as in a) but focusing on the comparison among regions for each mutation type. The number of derived alleles of each mutation type per region (colour coded) as a violin plot. Individual values are shown as dots. The median is shown as a horizontal line in each violin plot. The mean and its 95%CI of each distribution are shown as a coloured square with their corresponding error bars. The sample sizes of individuals for which summary statistics are derived from, together with other statistics, are indicated in Supplementary Table 10.

Region	Number of samples	Derived allele accumulation	
		mean	SE
West Eurasia	71	31,408.11	62.55
South Asia	39	31,418.14	55.82
America	20	31,418.16	83.02
Central Asia Siberia	27	31,074.56	88.84
East Asia	45	31,070.03	81.18

Supplementary Table 9. Derived allele accumulation per region. Summary statistics of the derived allele accumulation per region (Methods, S8). For each region, the mean and its SE (Methods) are provided.

Region	Number of samples	T					
		T>A		T>C		T>G	
		mean	SE	mean	SE	mean	SE
West Eurasia	71	2,121.03	8.08	8,945.03	18.61	2,238.92	15.62
South Asia	39	2,178.67	10.37	9,052.31	18.86	2,249.06	10.87
America	20	2,279.48	13.65	9,073.85	22.32	2,340.38	36.87
Central Asia Siberia	27	2,237.16	12.38	8,998.01	30.52	2,257.20	31.05
East Asia	45	2,263.40	10.88	9,009.67	25.47	2,305.84	17.53

Region	Number of samples	C					
		C>A		C>T		C>G	
		mean	SE	mean	SE	mean	SE
West Eurasia	71	2,812.87	7.97	12,596.73	27.85	2,693.50	6.98
South Asia	39	2,876.78	8.79	12,333.88	31.13	2,727.45	8.86
America	20	2,909.30	14.33	12,077.40	27.41	2,737.03	9.51
Central Asia Siberia	27	2,851.09	13.00	12,047.83	46.08	2,683.32	8.41
East Asia	45	2,891.86	11.60	11,911.48	34.25	2,687.79	10.42

Region	Number of samples	C					
		CpG>TpG		T>C’		TCC>TTC	
		mean	SE	mean	SE	mean	SE
West Eurasia	71	4,094.58	11.84	7,684.90	17.16	817.33	4.42
South Asia	39	4,103.72	11.79	7,528.18	19.02	701.84	8.43
America	20	4,094.31	14.92	7,353.33	18.34	629.97	4.82
Central Asia Siberia	27	4,064.08	19.52	7,341.55	27.96	642.08	10.48
East Asia	45	4,067.15	16.60	7,252.85	19.85	591.16	4.47

Supplementary Table 10. Derived allele accumulation per region stratified per mutation type. Summary statistics of the derived allele accumulation per region for each mutation type (Methods, S8). For each region and mutation type, the mean and its SE (Methods) are provided.

S9 - Estimation of the different parental generation time in West Eurasia and East Asia

As described in the main text, West Eurasia individuals have accumulated 1.09% more derived alleles than East Asians since the split with Africans (Out-of-Africa). Because we are only interested in the proportion of derived alleles accumulated after the split of West Eurasians and East Asians, we need to correct for the span of time since the Out-of-Africa event until the split of the two Eurasian populations (Supplementary Figure 13). Thus, we need to assume dates for the split between Africans and non-Africans and the split between Eurasians.

We note that in the literature dating the Out-of-Africa is widely discussed and controversial, since it was not a clean split between non-Africans and Africans. Instead, from MCMC results and cross coalescence rate analysis in Bergström et al. 2020[13] and in Schiffels and Durbin 2014[27] the authors note that there might have been a gradual separation among African populations and between Africans and non-Africans. They suggest that this process created population structure between 200,000 - 100,000 years ago within Africa and that the non-African group had more gene flow with certain African groups (i.e., Yorubans) than others (i.e., San). After that, the rate increased, indicative of an accelerated split between Africans and non-Africans which has the median divergence point between 80,000 - 60,000 years ago. Similarly, the split among Eurasians was not clean either. All splits started around 70,000 years ago with a median divergence point between 40,000 and 20,000 years ago for East Asians and West Eurasians. Nonetheless, studies of ancient DNA show that around 40,000 years ago East Asians and West Eurasians were already diverging: the ancient human sample of Kostenki (36,000 year old sample) presents higher affinity to present day West Eurasians[28] and Tianyuan (40,000 year old sample) to East Asians[29].

In this analysis, we assume that the split between Africans and non-Africans happened 60,000 years ago and that the split between West Eurasians and East Asians happened 40,000 years ago. This is because if the proportion of time the West Eurasians and East Asians were apart decreases in respect of the time since both splited from Africans (i.e., Out-of-Africa happening 80,000 instead of 60,000 years ago), the rate at which mutations should have accumulated would have been higher. Thus, a conservative measurement will be assuming a lower bound for the Out-of-Africa.

Following the conversion of percent excess of derived alleles (1.09%) into differences in mean generation time outlined in the Methods section, we estimate that generation times in East Asians have been 2.68 to 3.39 years longer than in West Eurasians since the split of the two

populations. This corresponds to West Eurasians having had approx. 150 generations more than East Asians.

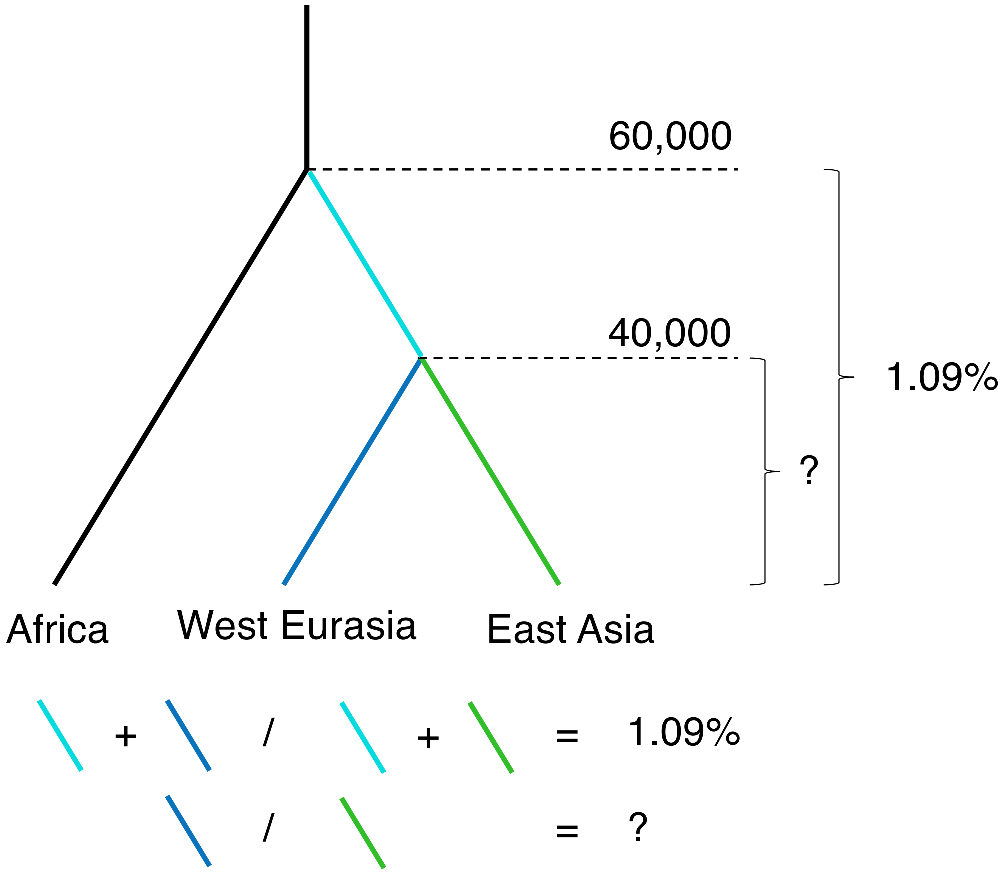

Supplementary Figure 13. Mutation rate difference between West Eurasia and East Asia. This diagram shows conceptually that the mutation rate could only be different after the split between East Asians and West Eurasians (blue and green terminal branches). However, the difference in derived allele accumulation is calculated since the split with Africans for each group (cyan and blue, cyan and green).

S10 - Mutation spectrum correlation with mean parental age and potential bias due to difference in mean paternal and maternal age in de deCODE dataset

The germline mutation spectrum is dependent on the parental sex and age at conception[30]. In this study, we observe differences in the abundance of derived alleles accumulated after the Out-of-Africa event when stratified by mutation type (Supplementary Figure 12, Supplementary Table 10). Here, we study to which extent these differences can be explained by changes in generation time in the 5 regions. For that, we compare the mutational patterns of *de novo* mutations (DNM) depending on parental age in trio studies[30,31] (deCODE data set) with the differences in mutation spectrum of extant populations with the mean archaic fragment length as a proxy of mean generation time (SGDP data set) as explained in the Methods section.

The estimates of the obtained linear models for each mutation type are given in Supplementary Table 11. Moreover, the correlations between the slopes of both data sets is shown in Supplementary Figure 14.

The probands of the deCODE data set have a bias towards fathers being older than mothers, with a mean of 2.77 years and the largest difference of more than 40 years (Supplementary Figure 15a). To study if the correlation of the mutation spectrum with the mean parental age is affected by the mentioned bias, we rerun the correlation test with the deCODE data set with only probands that have parents with an age difference of less than 4 years. This way, we retaining more than 50% of the data (Supplementary Figure 15b) and reduce the bias (mean = 0.94 differences in years, Supplementary Figure 15c). We then compared the slopes of the linear models calculated in the original deCODE data set and when we impose the parental age difference filter explained above (Supplementary Figure 16). We don’t observe qualitative changes in the slopes when comparing the two and thus, we used all probands for our analysis.

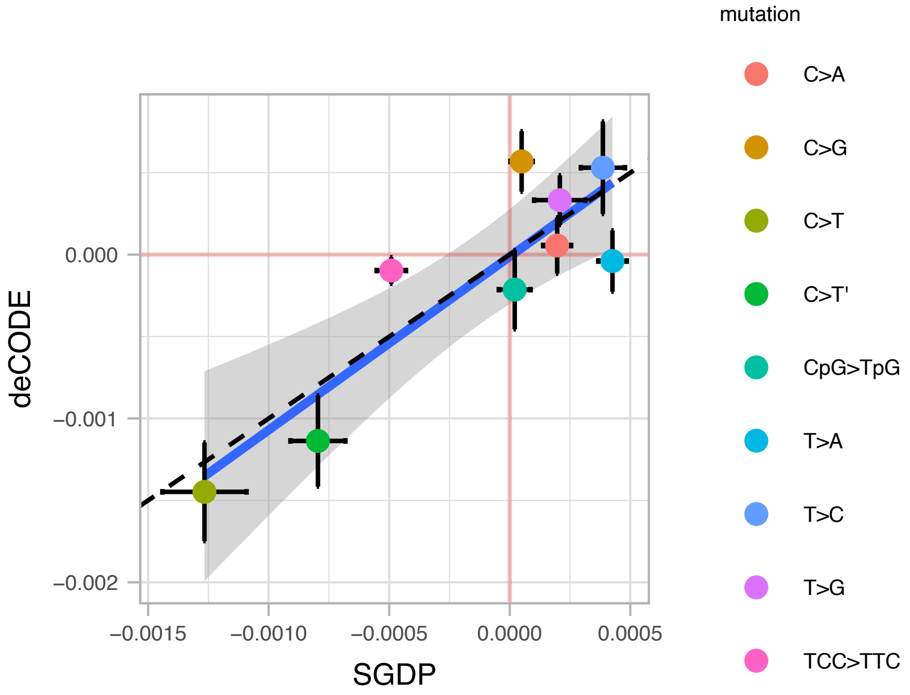

Supplementary Figure 14. Slope coefficient correlation between SGDP data and deCODE data linear models. Dot plot graph illustrating the correlation between linear model slope coefficients derived from the SGDP data (x-axis) and deCODE data (y-axis) for each mutation type (color code). 95%CI for each estimate are shown as error bars. The sample sizes of individuals for which summary statistics are derived from, together with other statistics, are indicated in Supplementary Table 2, Supplementary Table 10, Supplementary Table 11 and S10. The 1-to-1 correspondence is denoted by the black dashed diagonal line. The linear model between data sets' slopes is shown as a blue line with it's 95%CI as a shaded region around it. Linear model : deCODE slope = -1.196e-05 + 1.058 * SGDP slope. F-test, P value = 1.768e-3. Adjusted R^2 = 0.7414.

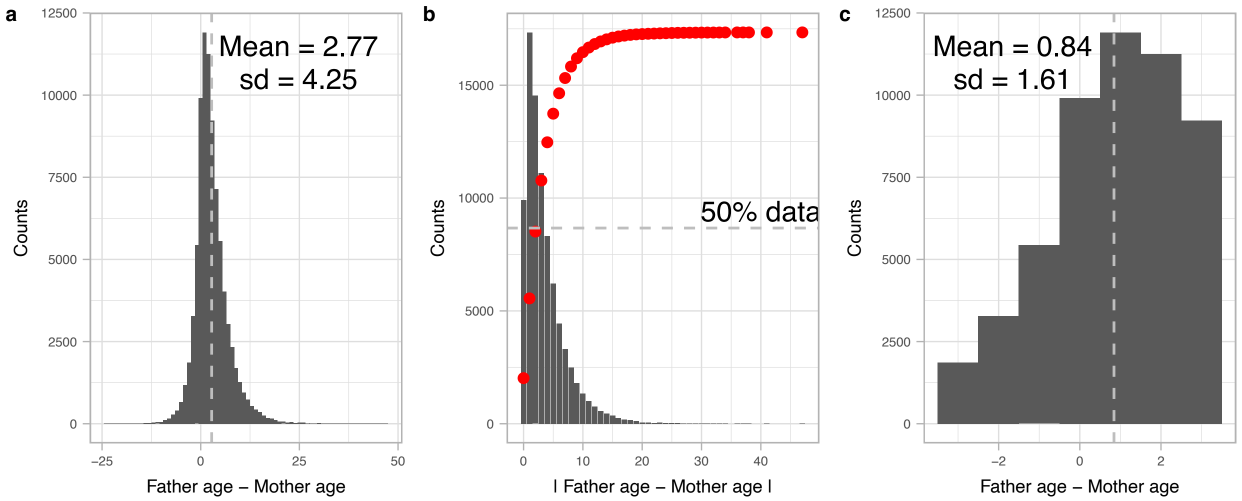

Supplementary Figure 15. Parental age difference in the deCODE data. a) Histogram of the number of probands with a certain parental age difference. The mean is shown as a vertical gray line and annotated as a numeric figure. **b)** Histogram of the number of probands with a certain absolute parental age difference. The cumulative distribution of provands is denoted by red dots. The horizontal gray line shows the 50% data threshold. **c)** Histogram of the number of probands with a certain parental age difference with less than 4 years difference. The mean is shown as a vertical gray line and annotated as a numeric figure.

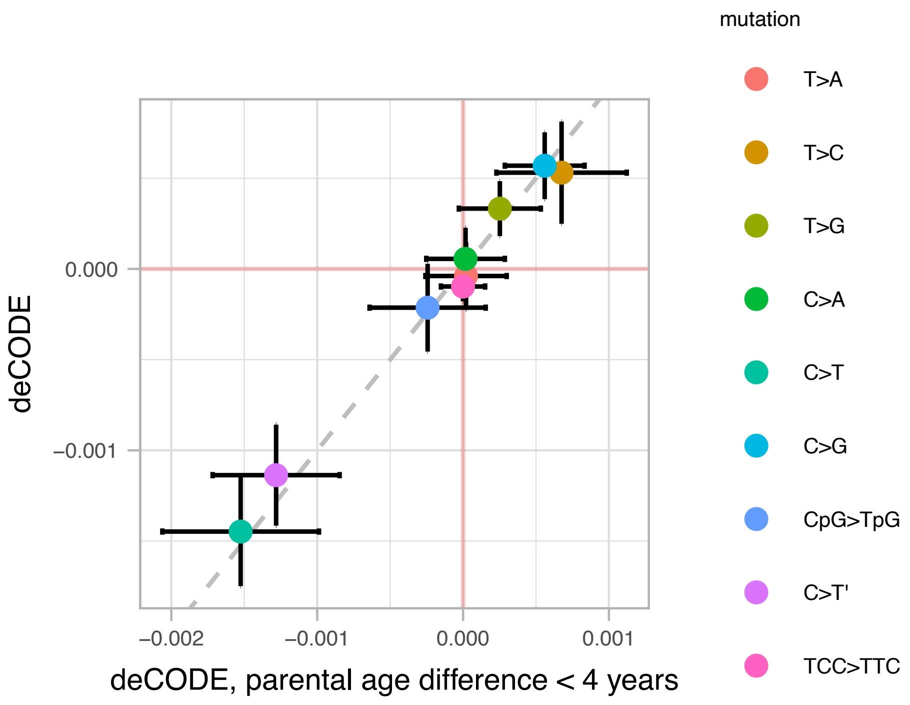

Supplementary Figure 16. Slope coefficient correlation between linear models of deCODE data and deCODE data when only using probands with parental age difference less than 4 years. Dot plot graph illustrates the correlation between linear model slope coefficients derived from the deCODE data (y-axis) and the deCODE data when only using probands with parental age difference less than 4 (x-axis) for each mutation type (color code). 95%CI for each estimate are shown as error bars. The sample sizes of individuals for which summary statistics are derived from, together with other statistics, are indicated in Supplementary Table 11 and S10. The 1-to-1 correspondence is denoted by the gray dashed diagonal line.

Mutation	Data set	Intercept	SE	t value	P value
T>A	deCODE	6.72e-2	2.86e-3	23.47	9.70e-34
	SGDP	3.71e-2	2.34e-3	15.85	3.40e-37
T>C	deCODE	2.46e-1	4.33e-3	56.79	9.27e-58
	SGDP	2.58e-1	3.63e-3	71.00	8.39e-144
T>G	deCODE	5.34e-2	2.33e-3	22.98	3.34e-33
	SGDP	5.64e-2	4.22e-3	13.36	1.66e-29
C>A	deCODE	8.79e-2	2.63e-3	33.43	4.26e-43
	SGDP	7.61e-2	2.29e-3	33.19	2.77e-83
C>T	deCODE	4.69e-1	4.62e-3	101.48	3.35e-74
	SGDP	4.90e-1	6.92e-3	70.86	1.22e-143
C>G	deCODE	7.70e-2	2.83e-3	27.21	1.37e-37
	SGDP	8.25e-2	1.83e-3	45.16	7.19e-107
CpG>TpG	deCODE	1.82e-1	3.71e-3	48.96	1.32e-53
	SGDP	1.29e-1	2.62e-3	49.30	7.50e-114
C>T'	deCODE	2.65e-1	4.27e-3	62.02	3.08e-60
	SGDP	3.01e-1	4.52e-3	66.54	2.14e-138
TCC>TTC	deCODE	2.19e-2	1.25e-3	17.54	1.55e-26
	SGDP	6.05e-2	2.41e-3	25.13	8.48e-64

Mutation	Data set	Slope	SE	t value	P value
T>A	deCODE	-3.92e-5	9.52e-5	-0.41	6.82e-1
	SGDP	4.26e-4	3.02e-5	14.13	7.04e-32
T>C	deCODE	5.30e-4	1.44e-4	3.69	4.61e-4
	SGDP	3.86e-4	4.68e-5	8.27	1.91e-14
T>G	deCODE	3.32e-4	7.73e-5	4.30	5.77e-5
	SGDP	2.08e-4	5.44e-5	3.82	1.78e-4
C>A	deCODE	5.55e-5	8.74e-5	0.63	5.28e-1
	SGDP	1.97e-4	2.95e-5	6.66	2.60e-10
C>T	deCODE	-1.45e-3	1.54e-4	-9.43	7.52e-14
	SGDP	-1.27e-3	8.91e-5	-14.21	3.85e-32
C>G	deCODE	5.69e-4	9.41e-5	6.05	7.66e-8
	SGDP	4.92e-5	2.35e-5	2.09	3.77e-2
CpG>TpG	deCODE	-2.14e-4	1.23e-4	-1.73	8.82e-2
	SGDP	2.07e-5	3.37e-5	0.61	5.41e-1
C>T'	deCODE	-1.14e-3	1.42e-4	-8.01	2.58e-11
	SGDP	-7.96e-4	5.82e-5	-13.67	1.81e-30
TCC>TTC	deCODE	-9.72e-5	4.15e-5	-2.34	2.22e-2
	SGDP	-4.91e-4	3.10e-5	-15.84	3.79e-37

Supplementary Table 11. Linear models between mutation type fraction and mean generation time estimate in the SGDP and deCODE data sets. Two separate tables are given for the intercept and the slope of the linear models obtained using the R function `lm()`. For each mutation type and data set, the coefficients estimate, the SE, the t-test t value and the associated P value are provided.

S11 - Sex Specific mutational patterns

X-to-A ratio

Due to the inheritance pattern of the X chromosome - 2 copies transmitted in females while only 1 in males - compared to autosomes - 2 copies in both females and males -, it is expected that the X chromosome has ¾ the diversity of the autosomes. However, this can be altered if the mutation rate changes disproportionately between females and males due to shifts in generation time between sexes. For example, an increase in the male mean generation time will decrease the yearly mutation rate in males and thus, proportionally less mutations are going to be accumulated in autosomes compared to the X chromosomes[32]. Therefore, the ratio of derived allele accumulation between the X chromosome and the autosomes will reflect variation on the generation time between males and females: higher values of the X-to-A ratio will be indicative of longer generation times in males compared to females and vice versa. Although here we only consider generation time differences to affect the ratio, there are other factors that can perturbe this ratio such as reproductive variance between sexes[33], demographic events[34] or differences in selection[35].

We computed the X-to-A ratio as explained in the Methods section and we then correlated the ratio with the mean archaic fragment length for each individual (Figure 4a).

C>G maternaly enriched regions

As described in Jónsson et al. 2017[30], there are regions of the genome in which DNM are clustered (cDNM). Those regions appear to be enriched in C>G mutations which originate in the maternal lineage. They also show that these clusters increase in number more rapidly with maternal than paternal age at conception.

Here we explore if there is a difference in the number of C>G segregating sites in cDNM genomic windows among the 5 regions.

To compute the C>G ratio between DNM cluster regions and the rest of the genome, we follow the procedure explained in the Methods section.

If the ratio r = 1, it indicates that the C>G enrichment is similar in cDNM regions compared to the rest of the genome. If r > 1, then there is an excess and if r < 1, then there is a depletion. Nonetheless, we are not interested in the actual ratio, but the comparison among geographical

regions on this quantity. We then correlated the ratio with the mean archaic fragment length for each individual obtained in this study (Figure 4b).

Y chromosome

Male individuals with shorter generation time are predicted to increase the mutation rate per year. Thus, Y chromosomes are expected to accumulate more derived alleles in individuals with a historically shorter mean generation time compared to others with longer ones.

To investigate that, we followed a similar procedure as explained in the Methods and in S8 to classify derived alleles into mutation types, changing certain steps and filters listed below:

1. We only used males in SGDP data
2. Alleles were polarized using the Chimp sequence in human coordinates. Since the chimpanzee Y chromosome is not provided with the SGDP data, this was achieved by taking the chimpanzee sequence from the hg19-panTro6 alignment into a fasta file with the human coordinates. The alignment can be downloaded from the following link:

http://hgdownload.cse.ucsc.edu/goldenpath/hg19/vsPanTro6/reciprocalBest/axtRBestNet/hg19.panTro6.rbest.axt

3. No archaic regions were masked since there is no evidence of archaic sequence in the modern human Y chromosome
4. Only polymorphisms in the X degenerate regions are considered (coordinates from [36]) and no further filters regarding repetitive regions were imposed
5. Individuals S_Finnish-2, S_Finnish-3, S_Palestinian-2, S_Mansi-1 and S_Masai-2 were discarded from the analysis because they didn't yield any callable polymorphism
6. For each individual, all heterozygous sites were classified as non-callable sites
7. Only African individuals with Y haplogroups A and B (metadata provided in [1], A: S_Ju_hoan_North-2, S_Dinka-2; B: S_Biaka-1, S_Biaka-2, S_Mbuti-3, S_Ju_hoan_North-3, S_Ju_hoan_North-1) were used as the outgroup. If polymorphisms were found to be segregating in these individuals, they were filtered out from this analysis
8. We didn't require the 5' and 3' contiguous base pairs (context) of a polymorphic site to be callable

The accumulation of derived alleles in the Y chromosome per geographical region is shown in Supplementary Figure 17 (included in Data2_mutationspectrum.txt) and in Supplementary Table 13.

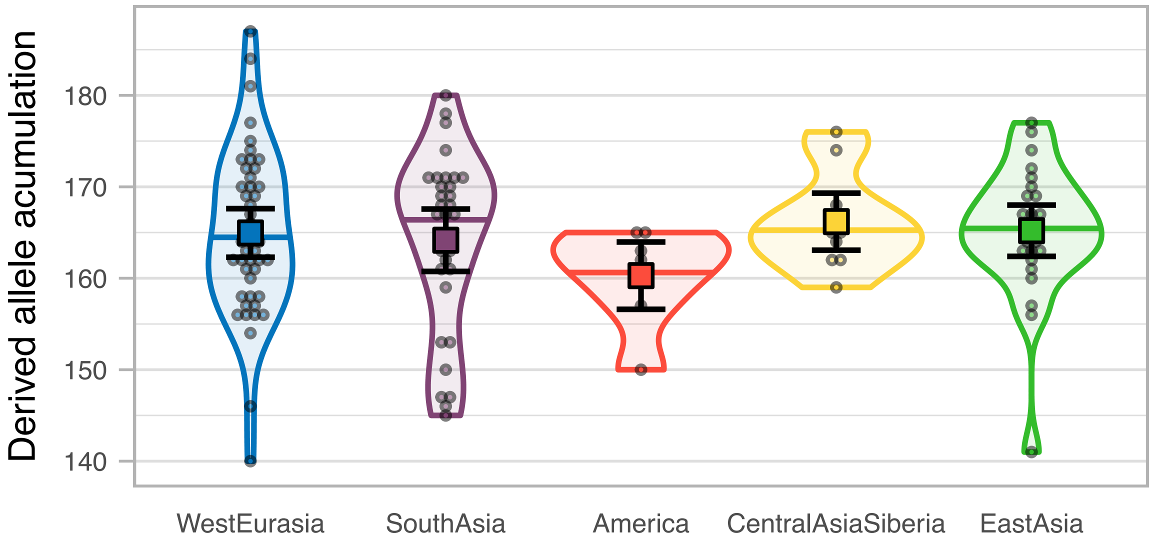

Supplementary Figure 17. Mean derived allele accumulation per region in the Y Chromosome. The number of derived alleles of each mutation type per region (colour coded) as a violin plot. Individual values are shown as dots. The median is shown as a horizontal line in each violin plot. The mean and its 95%CI of each distribution are shown as a coloured square with their corresponding error bars. The sample sizes of individuals for which summary statistics are derived from, together with other statistics, are indicated in Supplementary Table 13.

Region	Number of samples	Derived allele accumulation (X chromosome)	
		mean	SE
West Eurasia	23	2,820.38	21.20
South Asia	8	2,911.27	26.60
America	13	2,839.27	21.59
Central Asia Siberia	16	2,818.79	18.54
East Asia	20	2,900.52	17.36

Supplementary Table 12. Derived allele accumulation per region for the X chromosome in female individuals. Summary statistics of the derived allele accumulation per region on the X chromosome of females. For each region, the mean and its SE (Methods) are provided.

Region	Number of samples	Derived allele accumulation (Y chromosome)	
		mean	SE
West Eurasia	45	164.96	1.35
South Asia	31	164.16	1.74
America	7	160.29	1.88
Central Asia Siberia	10	166.19	1.59
East Asia	25	165.20	1.43

Supplementary Table 13. Derived allele accumulation per region for the Y chromosome in male individuals. Summary statistics of the derived allele accumulation per region on the X chromosome of males. For each region, the mean and the SE (Methods) are provided.

S12 - Source Data

Data1_archaicfragments.txt: Archaic fragments found in individuals from the 5 main geographical regions and ancient samples in the SGDP investigated in this study. Each line is a fragment with the following attributes:

1. name: individual the fragment belongs to.
2. region: region that the individual belongs to as defined by Mallick et al. 2016[1].
3. chrom: chromosome in which the fragment is located.
4. start: starting fragment position in hg19 coordinates.
5. end: ending fragment position in hg19 coordinates.
6. length: fragment length (end - start).
7. MeanProb: mean posterior probability for the fragment outputted by the Skov et al. 2018 method[2].
8. snps: number of SNPs found in the fragment that are not segregating in any of the Sub-Saharan African genomes (S1).
9. Altai: number of SNPs found in the fragment that are shared with the Altai Neanderthal[17].
10. Denisova: number of SNPs found in the fragment that are shared with the Denisova[18].
11. Vindija: number of SNPs found in the fragment that are shared with Vindija Neanderthal[12].

Data2_mutationspectrum.txt: Counts of derived alleles classified into the 96 mutation types for the extant samples of the SGDP, per chromosome. Each line has the following attributes:

1. ind: individual identifier
2. reg: region that the individual belongs to as defined by Mallick et al. 2016[1].
3. sex: individual sex defined by Mallick et al. 2016[1]. M = male, F = female.
4. chrom: chromosome which the counts belong to.
5. fiv: contiguous 5' base pair of the focal SNP
6. anc: ancestral allele of the mutation
7. thr: contiguous 3' base pair of the focal SNP
8. der: ancestral allele of the mutation
9. counts: number of mutation types found

Data3_HGDParchaicfragments.txt: Archaic fragments found in individuals from the 4 populations assessed from the HGDP data set investigated in this study. Each line is a fragment with the following attributes:

1. name: individual the fragment belongs to.
2. population: population that the individual belongs to as defined by Bergström et al. 2020[13].
3. region: region that the individual belongs to as defined by Bergström et al. 2020[13].
4. chr: chromosome in which the fragment is located.
5. start: starting fragment position in hg38 coordinates.
6. end: ending fragment position in hg38 coordinates.
7. length: fragment length (end - start).
8. snps: number of SNPs found in the fragment that are not segregating in any of the Sub-Saharan African genomes (S1).
9. meanprob: mean posterior probability for the fragment outputted by the Skov et al. 2018 method[2].

References

1. Mallick, S. *et al.* The Simons Genome Diversity Project: 300 genomes from 142 diverse populations. *Nature* **538**, 201–206 (2016).
2. Skov, L. *et al.* Detecting archaic introgression using an unadmixed outgroup. *PLoS Genet.* **14**, e1007641 (2018).
3. Skov, L. *et al.* The nature of Neanderthal introgression revealed by 27,566 Icelandic genomes. *Nature* **582**, 78–83 (2020).
4. The 1000 Genomes Project Consortium. A global reference for human genetic variation. *Nature* **526**, 68–74 (2015).
5. Li, H. A statistical framework for SNP calling, mutation discovery, association mapping and population genetical parameter estimation from sequencing data. *Bioinformatics* **27**, 2987–2993 (2011).
6. Fu, Q. *et al.* Genome sequence of a 45,000-year-old modern human from western Siberia. *Nature* **514**, 445–449 (2014).
7. Sikora, M. *et al.* The population history of northeastern Siberia since the Pleistocene. *Nature* **570**, 182–188 (2019).
8. Sikora, M. *et al.* Ancient genomes show social and reproductive behavior of early Upper Paleolithic foragers. *Science* **358**, 659–662 (2017).
9. Rasmussen, M. *et al.* The genome of a Late Pleistocene human from a Clovis burial site in western Montana. *Nature* **506**, 225–229 (2014).
10. Lazaridis, I. *et al.* Ancient human genomes suggest three ancestral populations for present-day Europeans. *Nature* **513**, 409–413 (2014).
11. Skoglund, P. & Jakobsson, M. Archaic human ancestry in East Asia. *Proc. Natl. Acad. Sci. U. S. A.* **108**, 18301–18306 (2011).
12. Prüfer, K. *et al.* A high-coverage Neandertal genome from Vindija Cave in Croatia. *Science* **358**, 655–658 (2017).
13. Bergström, A. *et al.* Insights into human genetic variation and population history from 929 diverse genomes. *Science* **367**, eaay5012 (2020).

14. Marchi, N., Winkelbach, L., Schulz, I. & Brami, M. The mixed genetic origin of the first farmers of Europe. *bioRxiv* (2020).
15. Mathieson, I. *et al.* The genomic history of southeastern Europe. *Nature* **555**, 197–203 (2018).
16. Byrska-Bishop, M., Evani, U. S., Zhao, X. & Basile, A. O. High coverage whole genome sequencing of the expanded 1000 Genomes Project cohort including 602 trios. *bioRxiv* (2021).
17. Prüfer, K. *et al.* The complete genome sequence of a Neanderthal from the Altai Mountains. *Nature* **505**, 43–49 (2014).
18. Meyer, M. *et al.* A high-coverage genome sequence from an archaic Denisovan individual. *Science* **338**, 222–226 (2012).
19. Villanea, F. A. & Schraiber, J. G. Multiple episodes of interbreeding between Neanderthal and modern humans. *Nat Ecol Evol* **3**, 39–44 (2019).
20. Vernot, B. *et al.* Excavating Neandertal and Denisovan DNA from the genomes of Melanesian individuals. *Science* **352**, 235–239 (2016).
21. Wall, J. D. *et al.* Higher levels of neanderthal ancestry in East Asians than in Europeans. *Genetics* **194**, 199–209 (2013).
22. Kelleher, J., Etheridge, A. M. & McVean, G. Efficient Coalescent Simulation and Genealogical Analysis for Large Sample Sizes. *PLoS Comput. Biol.* **12**, e1004842 (2016).
23. Higham, T. *et al.* The timing and spatiotemporal patterning of Neanderthal disappearance. *Nature* **512**, 306–309 (2014).
24. Moorjani, P., Amorim, C. E. G., Arndt, P. F. & Przeworski, M. Variation in the molecular clock of primates. *Proc. Natl. Acad. Sci. U. S. A.* **113**, 10607–10612 (2016).
25. Harris, K. Evidence for recent, population-specific evolution of the human mutation rate. *Proc. Natl. Acad. Sci. U. S. A.* **112**, 3439–3444 (2015).
26. Harris, K. & Pritchard, J. K. Rapid evolution of the human mutation spectrum. *Elife* **6**, (2017).

27. Schiffels, S. & Durbin, R. Inferring human population size and separation history from multiple genome sequences. *Nat. Genet.* **46**, 919–925 (2014).
28. Seguin-Orlando, A. *et al.* Paleogenomics. Genomic structure in Europeans dating back at least 36,200 years. *Science* **346**, 1113–1118 (2014).
29. Fu, Q. *et al.* DNA analysis of an early modern human from Tianyuan Cave, China. *Proc. Natl. Acad. Sci. U. S. A.* **110**, 2223–2227 (2013).
30. Jónsson, H. *et al.* Parental influence on human germline de novo mutations in 1,548 trios from Iceland. *Nature* **549**, 519–522 (2017).
31. Halldorsson, B. V. *et al.* Characterizing mutagenic effects of recombination through a sequence-level genetic map. *Science* **363**, eaau1043 (2019).
32. Amster, G. & Sella, G. Life History Effects on Neutral Diversity Levels of Autosomes and Sex Chromosomes. *Genetics* **215**, 1133–1142 (2020).
33. Keinan, A., Mullikin, J. C., Patterson, N. & Reich, D. Accelerated genetic drift on chromosome X during the human dispersal out of Africa. *Nat. Genet.* **41**, 66–70 (2009).
34. Amster, G., Murphy, D. A., Milligan, W. R. & Sella, G. Changes in life history and population size can explain the relative neutral diversity levels on X and autosomes in extant human populations. *PNAS* **117**, 20063–20069 (2020).
35. Hammer, M. F. *et al.* The ratio of human X chromosome to autosome diversity is positively correlated with genetic distance from genes. *Nat. Genet.* **42**, 830–831 (2010).
36. Skov, L., Danish Pan Genome Consortium & Schierup, M. H. Analysis of 62 hybrid assembled human Y chromosomes exposes rapid structural changes and high rates of gene conversion. *PLoS Genet.* **13**, e1006834 (2017).
