# Supplementary material for: Different historical generation intervals in human populations inferred from Neanderthal fragment lengths and mutation signatures
Source: Nat Commun. 2021 Sep 7;12:5317. doi: 10.1038/s41467-021-25524-4 (PMC8423828; doi:10.1038/s41467-021-25524-4)
Supplement: Supplementary file 3 — Description of Additional Supplementary Files [file 41467_2021_25524_MOESM3_ESM.pdf]

Description of Additional Supplementary Files

Supplementary Data 1: Description

Archaic fragments found in individuals from the 5 main geographical regions and ancient samples in the SGDP investigated in this study. Each line is a fragment with the following attributes:

1. name: individual the fragment belongs to.
2. region: region that the individual belongs to as defined by Mallick et al. 2016.
3. chrom: chromosome in which the fragment is located.
4. start: starting fragment position in hg19 coordinates.
5. end: ending fragment position in hg19 coordinates.
6. length: fragment length (end - start).
7. MeanProb: mean posterior probability for the fragment outputted by the Skov et al. 2018 method.
8. snps: number of SNPs found in the fragment that are not segregating in any of the Sub-Saharan African genomes (S1).
9. Altai: number of SNPs found in the fragment that are shared with the Altai Neanderthal.
10. Denisova: number of SNPs found in the fragment that are shared with the Denisova.
11. Vindija: number of SNPs found in the fragment that are shared with Vindija Neanderthal.

Supplementary Data 2: Description

Counts of derived alleles classified into the 96 mutation types for the extant samples of the SGDP, per chromosome. Each line has the following attributes:

1. ind: individual identifier
2. reg: region that the individual belongs to as defined by Mallick et al. 2016.
3. sex: individual sex defined by Mallick et al. 2016. M = male, F = female.
4. chrom: chromosome which the counts belong to.
5. fiv: contiguous 5’ base pair of the focal SNP
6. anc: ancestral allele of the mutation
7. thr: contiguous 3’ base pair of the focal SNP
8. der: ancestral allele of the mutation
9. counts: number of mutation types found

Supplementary Data 3: Description

Archaic fragments found in individuals from the 4 populations assessed from the HGDP data set investigated in this study. Each line is a fragment with the following attributes:

1. name: individual the fragment belongs to.
2. population: population that the individual belongs to as defined by Bergström et al. 2020.
3. region: region that the individual belongs to as defined by Bergström et al. 2020.
4. chr: chromosome in which the fragment is located.
5. start: starting fragment position in hg38 coordinates.
6. end: ending fragment position in hg38 coordinates.
7. length: fragment length (end - start).
8. snps: number of SNPs found in the fragment that are not segregating in any of the Sub-Saharan African genomes (S1).
9. meanprob: mean posterior probability for the fragment outputted by the Skov et al. 2018 method.
